# Physiological Response of *Miscanthus x giganteus* to Plant Growth Regulators in Nutritionally Poor Soil

**DOI:** 10.3390/plants9020194

**Published:** 2020-02-05

**Authors:** Hana Malinská, Valentina Pidlisnyuk, Diana Nebeská, Anna Erol, Andrea Medžová, Josef Trögl

**Affiliations:** 1 Department of Biology, Faculty of Science, Jan Evangelista Purkyně University in Ústí nad Labem, Ústí nad Labem 400 96, Czech Republic; andrea.medzova@gmail.com (A.M.); ania.radziszewska@gmail.com (A.E.); 2 Department of Environmental Chemistry and Technology, Faculty of Environment, Jan Evangelista Purkyně University in Ústí nad Labem, Ústí nad Labem, 400 96, Czech Republic; pidlisnyuk@gmail.com (V.P.); diana.nebeska@ujep.cz (D.N.); josef.trogl@ujep.cz (J.T.); 3 Clinical Research Centre, Medical University of Białystok, Białystok, 15-089, Poland

**Keywords:** *Miscanthus x giganteus*, leaf fluorescence, nutritionally poor post-military soil, plant physiology, plant stress

## Abstract

*Miscanthus x giganteus* (Mxg) is a promising second-generation biofuel crop with high production of energetic biomass. Our aim was to determine the level of plant stress of Mxg grown in poor quality soils using non-invasive physiological parameters and to test whether the stress could be reduced by application of plant growth regulators (PGRs). Plant fitness was quantified by measuring of leaf fluorescence using 24 indexes to select the most suitable fluorescence indicators for quantification of this type of abiotic stress. Simultaneously, visible stress signs were observed on stems and leaves and differences in variants were revealed also by microscopy of leaf sections. Leaf fluorescence analysis, visual observation and changes of leaf anatomy revealed significant stress in all studied subjects compared to those cultivated in good quality soil. Besides commonly used *F_v_*/*F_m_* (potential photosynthetic efficiency) and P.I. (performance index), which showed very low sensitivity, we suggest other fluorescence parameters (like dissipation, *DIo*/*RC*) for revealing finer differences. We can conclude that measurement of leaf fluorescence is a suitable method for revealing stress affecting Mxg in poor soils. However, none of investigated parameters proved significant positive effect of PGRs on stress reduction. Therefore, direct improvement of soil quality by fertilization should be considered for stress reduction and improving the biomass quality in this type of soils.

## 1. Introduction

Plant biomass is a renewable source of energy and feedstock for bio products compliant with sustainability goals [1]. Cultivation of biomass on high quality agricultural soils is, however, controversial due to competition with food production. Thus, different types of marginal sites, incompatible with food production, are under consideration for biomass production. According to Gerwin et al. [2], 46% of area in Europe were identified as marginal for different reasons (low fertility, texture, pH, salinity, wetting or contamination) and 22.6% of these sites were determined to be suitable for biomass production.

Second-generation biofuel crops present suitable crops for these sites. One of the perspective crops is the perennial grass *Miscanthus x giganteus* (Mxg), a triploid hybrid (2n = 3x = 57) derived from two parental species *Miscanthus sinensis* and *Miscanthus sacchariflorus* [3]. Its closest relatives are other important crops like sorghum, sugarcane or the worldwide produced crop, maize. It proved to be very adaptable to different climate zones, its efficient C_4_ metabolism contributes to high yields of biomass within single vegetation season [4]. Production is stable for more than 20 years, except for the first and second years after planting [5]. Due to the high content of lignin and cellulose [6], it can also serve as feedstock for bio products. The hybrid is sterile and does not represent any danger of invasion being reproduced only through vegetative rhizomes. Growing Mxg can be beneficial in different ways simultaneously: it can serve as fast growing source of biomass [7], it is capable of improving soil quality, retaining moisture and preventing soil erosion. Mxg is tolerant to different types of contaminants [8,9] and it has been already investigated for biomass production on several marginal soils [10,11,12].

Production of such crops is highly affected by climate [13]. Under current, rapidly changing weather conditions, Mxg showed 59% increase of yield compared to maize in field trials [4]. Also, compared to other C_4_ cold tolerant biomass crops like switchgrass, Mxg was more than twice as productive [14]. Different miscanthus species vary in tolerance to various physical factors. According to physiological measurements (*F_v_*/*F_m_*), Mxg displayed the highest vitality under cold (together with *M. sacchariflorus*) [15] and drought (together with *M. sinensis*) [16] stress among other miscanthus species. Mxg physiological response was more sensitive to salinity and combination of salinity and drought than *M. sinensis* but more tolerant than *M. floridulus* [16]. Also novel hybrids were developed, which exhibited higher yields under drought, salinity and cold stress conditions than Mxg [17]. However, they are not yet commercially available so Mxg is currently still the most often used one.

Anyway, in marginal soils Mxg biomass yields are usually significantly lower, compared to good quality soils [18]. There are various ways to improve the productivity. One of them is the application of plant growth regulators (PGRs) for growth stimulation. In our previous study [19], we tested effect of PGRs Stimpo and Regoplant, with already proven positive effect on yield of other energetic crops [20], on Mxg biomass parameters (yield, height, number of stems and roots length) and metal uptake. The results were contradictory for different soils and PGRs. Additionally, unusually high uptake of biogenic metals Mn and Zn was determined in Mxg above-parts if grown in poor soil with low metals concentrations compared to good quality soil. It was hypothesized that this fact was connected with stress caused by poor soil characteristics and insufficient nutrients supply. For energetic purposes the increased uptake of metals to above-ground biomass is generally undesired, therefore a deeper look on the miscanthus stress was needed.

Many types of stress can directly or indirectly influence the level of plant fitness. As a result, main metabolic functions can be altered or suppressed and even green vigorously growing plants can undergo severe stress [21] because the most important process, photosynthesis, was influenced.

The common approaches for investigation of plant fitness level include analyses of secondary metabolites [22], measuring the level of plant hormones [23] and determination of leaf pigments [24]. When using these methods, a certain amount of plant tissue has to be destructed and analyzed. Furthermore, these methods are time-consuming, costly and may create an additional stress while harming the plant. As an alternative, non-destructive measurement of leaf fluorescence [25,26,27] can give a broader picture about different stages of photosynthesis process throughout vegetation season [28,29,30]. This approach is fast, less laborious and minimizes additional stress to the plant.

It is known [31], that yield of leaf fluorescence can give valuable information about efficiency of primary photochemistry and plant fitness [32,33,34,35,36]. Different plant physiology indexes are applied for identification and quantification of plant stress [37]. These “vitality indexes” usually focus on processes within primary photochemistry, however, they can result in slightly different values. There is little data about the most appropriate type of measurement for identification of stress in plants grown in the nutritionally poor soils, so it is not clear, which of these numerous indexes is the most appropriate.

The use of microscopy for measurement of leaf sections, thickness of leaves and stomatal density can give a broader idea about understanding the tissue structure [38]. Quantification of certain specific structures, like sclerenchyma cells, can also reveal substantial changes between plants. Sclerenchyma cells have very thick secondary walls, usually lignified or incrusted by silicon oxides. In monocotyledonous plants, they are aggregated in clusters forming “caps” around vascular bundles. These cells provide mechanical support, and due to the thick walls, they do not possess protoplast so they are not capable of mitotic division [39]. Therefore, their number is final. However, there were only few data when microscopy research was used for studying of miscanthus leaves [40]. In that study only differences of leaf structure of miscanthus varieties in response to cold were measured and authors did not elaborate any stress factor related to the soil in which the plant grew.

In the current study, the complex approach was applied which united non-destructive method of measurement of leaf fluorescence with supplementary microscopic measuring at harvest. The evaluation of the common plant physiology values/indexes *F_v_*/*F_m_* and Performance index were combined with less used indicators: *ET_0_*/*RC*, *TR_0_*/*RC*, *REo*/*RC*, *DIo*/*RC*, *V_j_*, *V_i_*, *F_0_*, *F_m_*, *F_v_*, *Fo*/*Fm*, *ABS*/*CS* (*CSo*, *CSm*), *T_fm_*, *TR_0_*/*CS* (*CSo*, *CSm*), *ET_0_*/*CS* (*CSo*, *CSm*), *DI_0_*/*CS* (*CSo*, *CSm*), *REo*/*CS* (*CSo*, *CSm*), which can help to detect minor changes in primary phase of photosynthesis [27,37,41]. That combination permitted the study of the effect more deeply and to evaluate impact of soil properties and treatment of crop by two PGRs on the physiology parameters during plant growth in nutritionally poor post-military marginal soils.

## 2. Materials and Methods

### 2.1. Soils

Real, poor quality soils, for the experiment were taken from two points at the former military airport Hradcany, marked as Hradcany 1 (H1), 50°37’31′31″ N, 14°43′23″ E and Hradcany 2 (H2), 50°37′26″ N, 14°44′49″ E. A more detailed explanation of site location and sampling procedure was done previously [19]. Generally, soil samples were taken from upper 30 cm and homogenized according to the standard procedure [42]. Certified industrial compost (C) from composting plant was used as standard soil for comparison. The compost was compliant with Czech standard ČSN 465735 as “suitable for agricultural and garden use”.

Agrochemical parameters of soils were examined in Crop Research Institute, Czech Republic laboratory in accordance with methodology compatible with International Organization for Standardization (ISO) or European Committee for Standardization (CEN) standards [43,44]. Briefly, pH was determined in suspension of soil and water. Available nutrients (P, K, Ca, Mg) were extracted using Mehlich 3 extraction protocol and determined with ICP-OES. Total nitrogen, sulfur and carbon were measured with elemental analyzer Vario MAX CNS/CN and humus content was calculated as carbon content multiplied by Welte’s coefficient 1.724.

Both Hradcany soils were regosols, sandy types of soil with low water retention capacity and prone to acidification. According to online estimated pedologic-ecological unit (BPEJ) catalogue [45] production potential of this area is very low (code 5.21.10). Agrochemical data (Table 1) confirmed low fertility and slight acidity of both Hradcany soils. Slightly higher concentration of available nutrients was detected for H2 but H1 had higher content of organic matter. Nevertheless, in both soils nutrients and humus content was low. On the other hand, C soil was slightly alkali but it provided enough nutrients and organic matter.

Another approach for soil characterization was evaluation of the state of soil microorganisms, which play an important role in soil functions and plant growth. The soil microbial communities were characterized using phospholipid fatty acids (PLFA) by method compliant with ISO/TS 29843-2 [46]. It consists of extraction of total lipids with chloroform-methanol-phosphate buffer mixture, separation of phospholipids on SPE silica columns, mild alkaline methanolysis with KOH and methanol and determination of fatty acids methylesters (FAME) using GC-MS. Microbial activity was assessed via basal soil respiration as production of CO_2_ per minute from 1 g of soil by NaOH reversed titration as described in details previously [47]. The value of PLFA_tot_, representing total living microbial biomass, was very low in Hradcany soils. The other PLFA parameters also indicated the influence of stress conditions: low ratios F/B PFLA and G+/G− PLFA [48]. All tested parameters were little more favorable in H1 compared to H2, anyway they were much higher in C soil with exception of F/B PLFA (Table 1).

### 2.2. Experiment Design

The experiment design was the same as described in previous work investigating biomass parameters [19]. Two rhizomes of Mxg “Rankova Zorya” prepared from three-year-old plants produced by the Institute of Bioenergy Crops and Sugar Beet, Ukraine, were planted in each 20 L pot with 1 kg of sand, 1 kg of ceramzite drainage and 10 kg of soil. The weight of rhizomes was 20 ± 2 g. The depth of plating was about 10 cm.

The experiment was carried out in the city of Ústí n. L., in university campus (50.6664 N, 14.0319 E) on the roof of the Faculty of Environment. Pots were placed on white gardening tarpaulin (see Supplementary Material, Figure S3). The climate is moderate, central-European (graphs with month average temperature, precipitation and light period in 2017 are in Supplementary Material, Figure S4). Plants were grown one vegetation season (April–November 2017) in real outdoor conditions (and irrigated as necessary to keep the substrate wet (2–3 times per week).

Plants growing in Hradcany soils, with exception of control, were treated with one of two commercially available PGRs, Stimpo and Regoplant, which were provided by Agrobiotech, Ukraine. Those substances include essential micronutrients, phytohormones and natural extracts that promote growth of bacteria in the soil. Publicly available characteristics of used PGRs are shown in Table 2. More detailed composition was not provided by the producer.

Treatment of plants by PGRs was done in two different ways: pre-soaking of rhizomes in 10 L of PGR solution for 12 h before planting and rhizomes pre-soaking with combination of additional spraying of above part biomass with 100 mL of PGR solution per pot. The control non-treated rhizomes (H1, H2) were soaked in distilled water for the same time as those treated with PGRs. The first spraying was performed when 3–4 leaves appeared; the second spraying was performed two weeks later. Plants grown in compost were planted without any treatment.

PGRs concentrations were selected according to producer recommendation. Contrary to previous work about effect on biomass [19] where different PGRs concentrations were tested, here we focused mainly on variants with the highest concentration to make the results presentation clearer. Concentrations used in this manuscript and number of replicates for each treatment are listed in Table 3. Data for lower concentrations are presented separately in supplementary material (Table S1).

### 2.3. Physiological Parameters

Measurement of leaf chlorophyll a fluorescence was performed using portable fluorimeter Handy PEA (Hansatech Instruments, UK). After 15 min of dark adaptation, low beam (50 μmol photons/m^2^/s) of actinic light was applied for 90 s. Afterwards, saturation pulse with intensity of 3500 µmol/m^2^/s (650 nm) was emitted by 3 LED diodes. The third youngest fully developed leaf was measured for each plant. Measurements were performed multiple times throughout vegetation season, in the same time of the day, mainly in the morning, to avoid distortion of data by changing temperature. Total number of measurements was 235. Due to the large number of individuals (57), measurement during summer season could not be performed within morning hours, therefore, all measurements from hot days were eliminated from this study.

Basic fluorescence parameters were measured: *F_0_* (minimal fluorescence intensity, initial fluorescence after application of saturation pulse, when all reaction centers of PSII are open) and *F_m_* (maximal level of fluorescence measured when all PSII reaction centers are closed). In addition, values in between these stages, like *F_j_*_,_ equal to fluorescence at 2 ms, F_i_ corresponding to fluorescence at 30 ms and *F_k_* describing fluorescence after 300 µs were analyzed. OLKJIP curves were reconstructed as means of fluorescence values recorded between 10 µs and 1 s.

Based on these values, other physiological parameters were calculated: *V_j_* (fluorescence intensity at 2 ms), *V_i_* (fluorescence intensity at 30 ms), *F_v_*/*F_m_* ratio (maximum quantum yield of primary photochemistry), ET_0_ (electron transported), DI_0_ (energy dissipated) and TR_0_ (energy trapped) per reaction centre (RC) or cross section (CS). More indexes were calculated as various combinations of previously mentioned. The detailed description of all 24 indexes used in this study can be found in Appendix A.

Additionally double normalization of transient part 50 µs–300 µs (*W_OK_*) was done using data measured with 10 µs steps according to Oukarroum [49] to visualize L-band with peak at 150 µs which it is not well visible at curve itself. Subsequently the non-treated plants fluorescence value was subtracted from the transients of Stimpo and Regoplant treated plants to receive difference transients (Δ*W_OK_*).

*W_OK_* = (*Ft* − *F_O_*)/(*F_K_* − *F_O_*)

### 2.4. Microscopy

Microscopy analysis was performed at the end of vegetation season in order to avoid additional plant stress. The second fully developed leaf from one plant grown in soil H1, H2 and one from H2_R250 × 250 was extracted 5 cm from leaf tip and compared with the same material harvested from plant grown in soil C. Then, 3 × 4 mm blocks of leaves were frozen and sliced using Leica CM 1100 freezing microtome to 12 µm thick slices, mounted in water and observed under inverted fluorescence microscope NIB-100F, Novel. Autofluorescence was recorded at excitation of 400–410 nm with barrier filter 455 nm using camera Eurekam 3.0 PLUS, BEL. Images were processed for brightness and contrast only using Image J software. Measurements of anatomical traits were performed using Scopelmage 9.0.

Number of stomata per square cm was counted on bottom side of leaf (3 measurements per each leaf). Bundle size was measured using “radius” tool in Scopelmage software, measuring three biggest vascular bundles. Sclerenchyma cells above and under these largest bundles were counted. Only cells with less than 50% of inner content were counted as “stone cells”. Leaf thickness and small bundle distance were measured multiple times (40 times per each leaf).

### 2.5. Statistical Analysis

The fluorescence data were processed using Microsoft Excel version 16 and Statistica version 13.3. There were 12 or less data points for each group available, so normal distribution could not be assumed. Therefore non-parametrical Kruskal-Wallis test was used for testing the difference among varieties and treatments [50].

Permuted radar charts were calculated based on Porter et al. [51] using Microsoft Excel software version 16.

## 3. Results

### 3.1. Soil Type Effect

**Figure 1:**
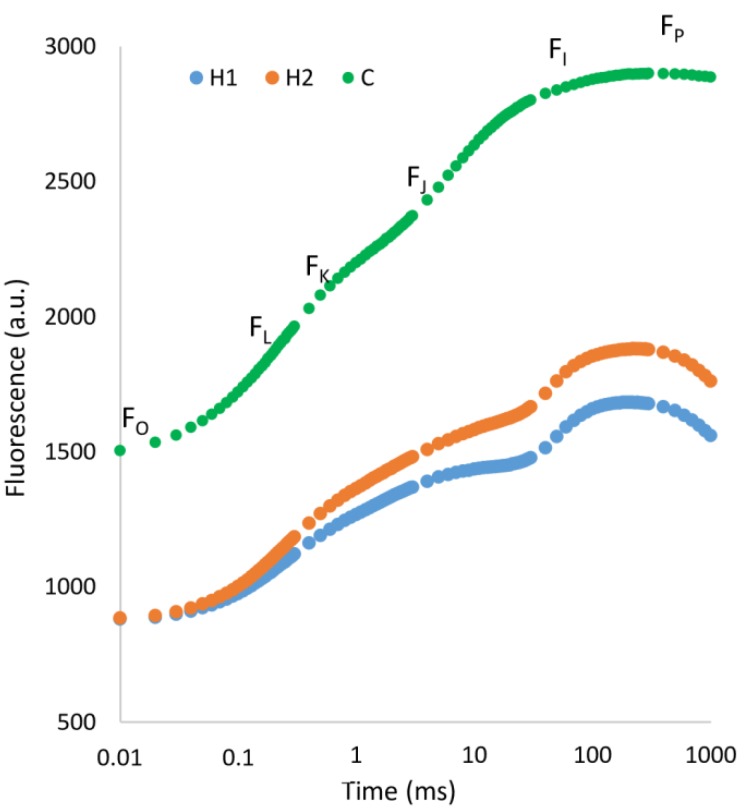
OLKJIP polyphasic fluorescence rise of plants grown in different types of soil calculated as mean value and plotted on a logarithmic time scale. Marks in graph refer to values used for calculation of certain parameters (Appendix A).

OLKJIP chlorophyll a induction curves were calculated for individuals grown in H1, H2 and C soil. It is obvious from Figure 1, C curve displays much higher values and steeper trajectory, more similar to classical OJIP as seen in literature [37].

**Figure 2:**
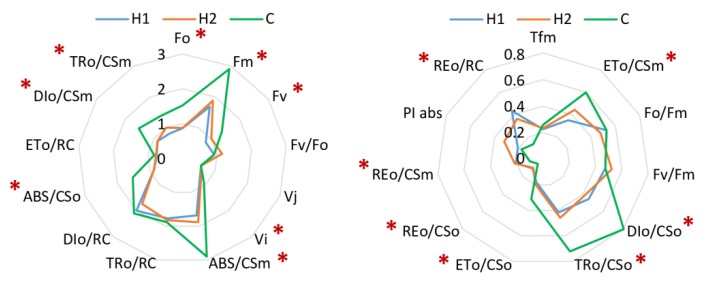
Physiological indexes of plants grown in different soils; red asterisks (*) marks indexes with significant differences between soils (*p* < 0.05). The data of different metrics were normalized for optimal presentation; original data can be found in supplementary Table S1.

For better comparison of values influenced by different factors, various fluorescence-based indexes were calculated (Figure 2). Based on 24 indexes calculated from data obtained from measurement of plants grown in compost (C), H1 and H2 without any treatment, we can conclude, that nutritionally rich soil (C, green line, Figure 2) provides better results in terms of height of the signals/parameters. Compared to C plants, H1 and H1 plants display much more similar values.

### 3.2. PGRs Treatments Effect

**Figure 3:**
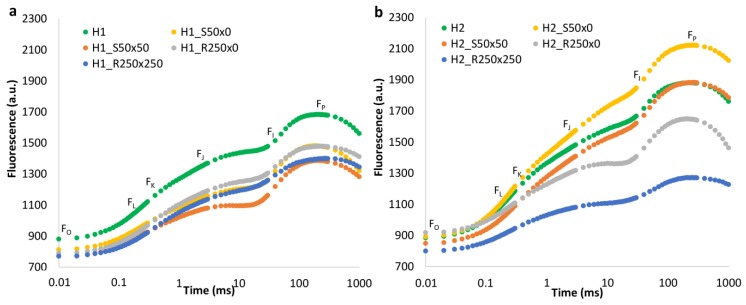
OLKJIP polyphasic fluorescence rise of plants grown in H1 (**a**) and H2 (**b**) supplemented by Stimpo and Regoplant calculated as mean value and plotted on a logarithmic time scale. Marks in graph refer to values used for calculation of certain parameters (Appendix A).

OLKJIP curves show different effect of PGRs on plants grown in different types of soil. In H1, Stimpo as well as Regoplant had very similar effect-decrease of fitness, which is also visible from decrease of fluorescence values and OLKJIP curve (Figure 3a). In H2, most treatments had negative influence on fluorescence level, mainly in the highest concentration of Regoplant (H2_R250 × 250). The only exception was in H2_S50 × 0 (Figure 3b).

Traditionally used parameters like *F_v_*/*F_m_* and P.I. showed partial differences between variants. Also, not so commonly used parameters like *Fo*/*Fm*, *DIo*/*CSo*, *TRo*/*CSo Tfm*, *ABS*/*CSm*, *DIo*/*RC* showed interesting differences among some groups. Decrease of *ABS*/*CSm* (compared to C plant) was observed in all treated and non-treated variants of H1 and H2. However, an increase of *DIo*/*RC* was only observed after the application of PGR. The strongest effect was after application of the highest concentration of Regoplant to individuals in soil H2. Parameters *DIo*/*CSo*, *ETo*/*CSo* and *ETo*/CSm showed statistical difference in treated and also non-treated plants.

**Figure 4:**
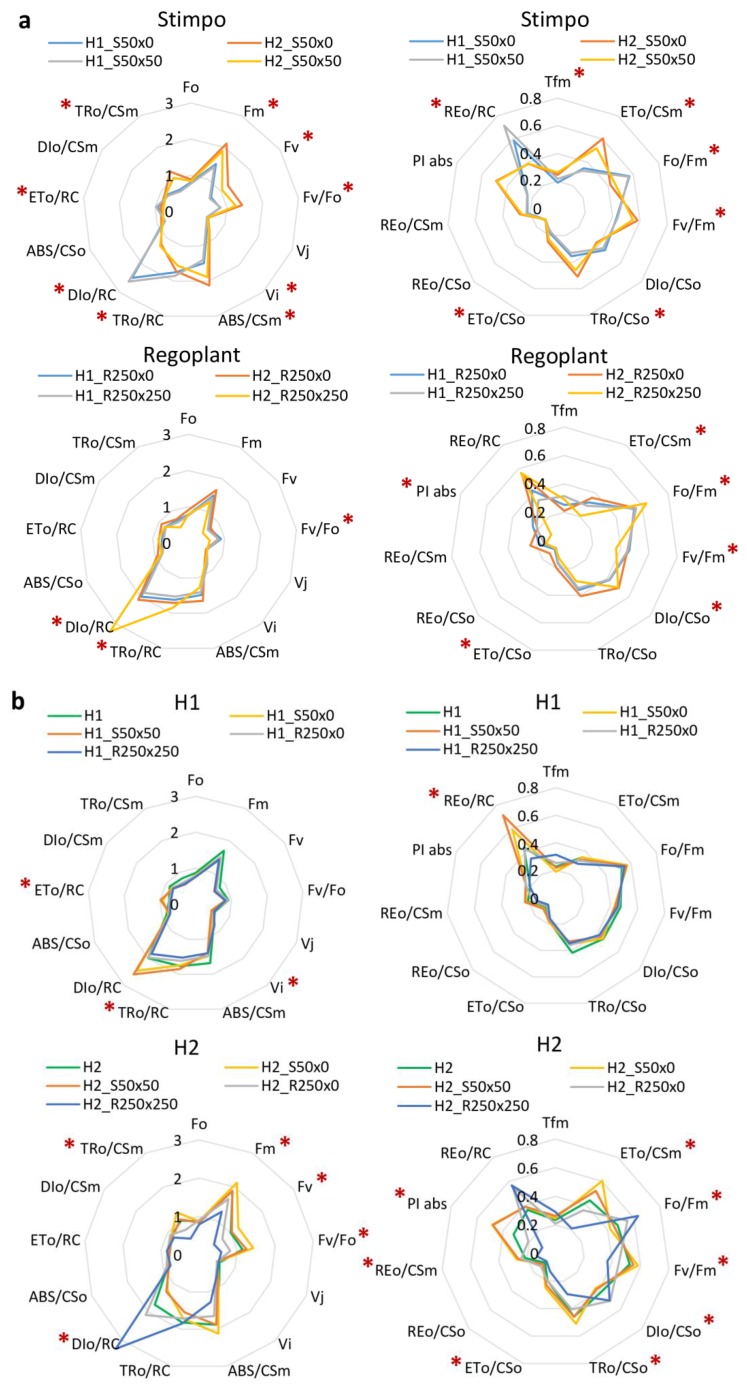
Physiological indexes for different PGRs treatment grouped by PGR (**a**) and soil (**b**); red asterisks (*) marks indexes with significant differences (*p* < 0.05). The data of different metrics were normalized for optimal presentation; original data can be found in Supplementary Table S1.

As seen in Figure 4, plants grown in two types of poor soil, H1 and H2 responded differently to the same concentration of PGR, independently on method of application. Stimpo treated plants displayed partial increase of certain parameters for H2 (*ETo*/*CSm*, *Fv*/*Fm*, *TRo*/*CSo*, *ABS*/*CSm*, *Fm*, *Fv*, *Fv*/*Fo* and P.I.; all values measured are summarized in Supplementary Material S2). The same parameters were usually decreased for H1 Stimpo treated plants. Moreover, H1 plants treated by Stimpo displayed elevated *Fo*/*Fm*, *REo*/*RC* and *DIo*/*RC*, whereas previously mentioned indexes were significantly lowered in H2. Increase of *Fv*/*Fm*, P.I. and some other parameters might imply certain positive effect of Stimpo, but mainly in H2 grown plants, not for H1 soil.

Regoplant treatment resulted in change of *DIo*/*RC* in all treated plants. In H2_R250 × 250, e.g., highest concentration of Regoplant, increase of this parameter was enormous, but not for H1 treated with the same concentration of stimulant. Again, the response of plants grown in different types of soil were different for the same treatment. Some minor changes occurred also for almost all other parameters, but not so evident.

**Figure 5:**
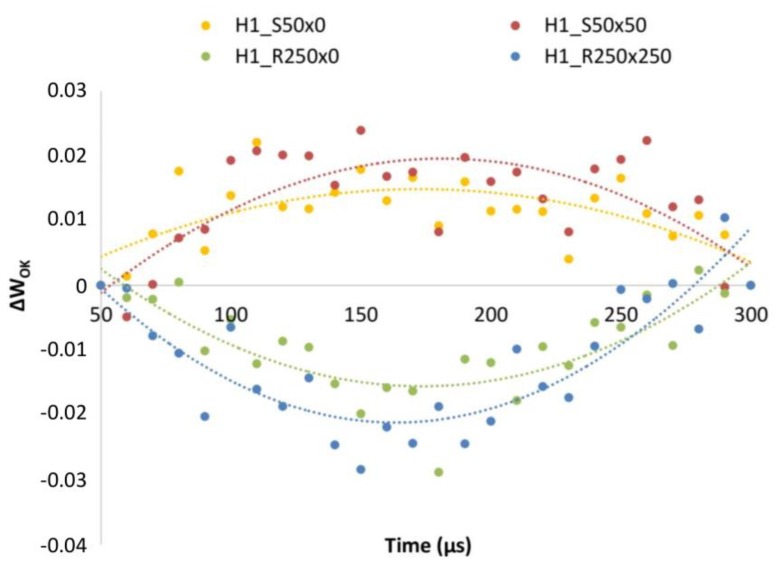
Fluorescence transient curves normalized between F_O_ and F_K_ for PGR treatment in H1 soil.

Due to the low resolution of JIP-test for H1 treated variants, we decided to take advantage of more sensitive method introduced recently [49], normalization of fluorescence signal between *F_o_* and *F_K_*. Figure 5 shows much clearer difference between H1 Stimpo and Regoplant treated plants compared to non-treated individuals (flat line, zero). This method reveals finer differences in the very beginning of the fluorescence curve, and seems to be very sensitive.

### 3.3. Effect on Morphology

**Figure 6:**
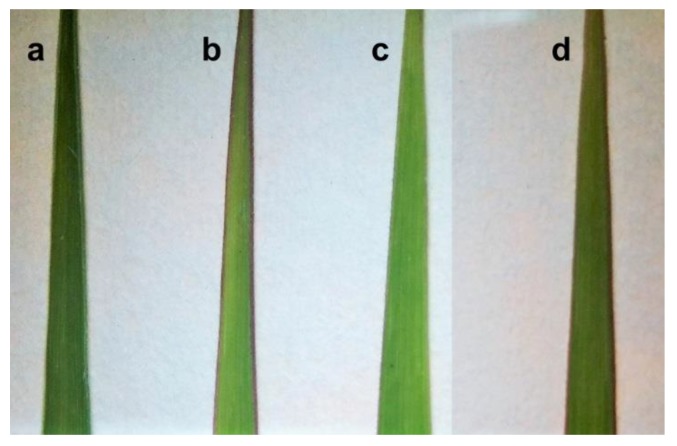
Photos of Mxg leaves used for microscopy sampled from variants (**a**) C, (**b**) H1, (**c**) H2 and (**d**) H2_R250 × 250.

**Figure 7:**
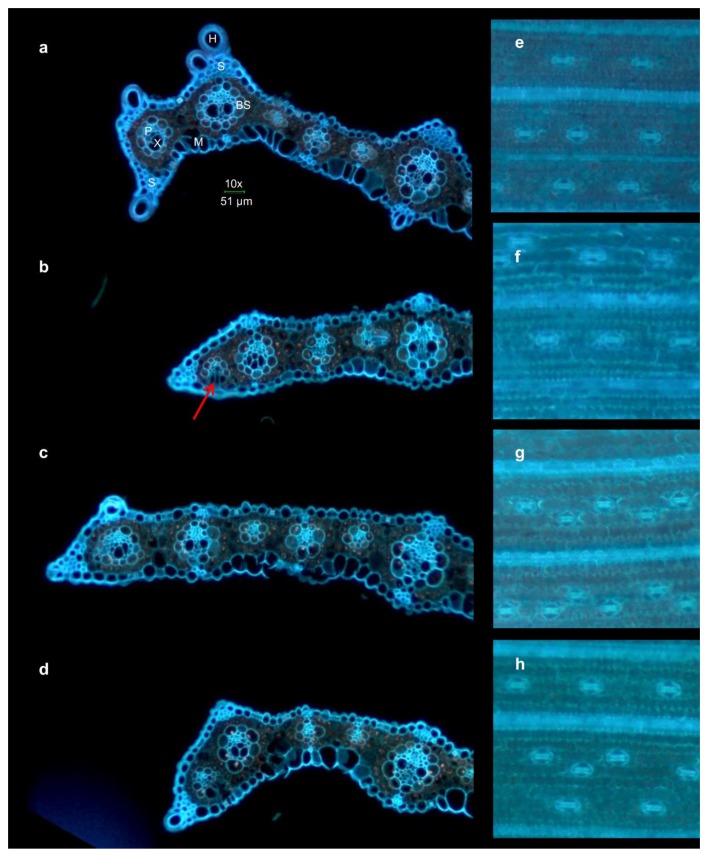
Fluorescence microscopy photos of Mxg leaves cross sections from experiment variants: C (**a**–**e**), H1 (**b**–**f**), H2 (**c**–**g**) and H2-R250 × 250 (**d**–**h**); leaves used for microscopy using autofluorescence (**a**–**d**), stomata on the bottom side of the leaf, autofluorescence (**e**–**h**); X-xylem, P-phloem, M-motor cell, S-sclerenchyma cell, H-hook, BS-bundle sheath cell, red arrow points altered bundle sheath.

The autofluorescence of leaf surface and transversal leaf sections confirmed severe changes in morphology of plants grown in the different soil types. The comparison of H2, H2_R250 × 250 plants with H1 plant and C plant is presented in Figure 6 and Figure 7. Leaves of C plants were green (Figure 6a) and vigorous, they had well organized stomata in one row, as seen from bottom of the leaf (Figure 7e) and they were rich in sclerenchyma cells as seen on transversal section (Figure 7).

**Figure 8:**
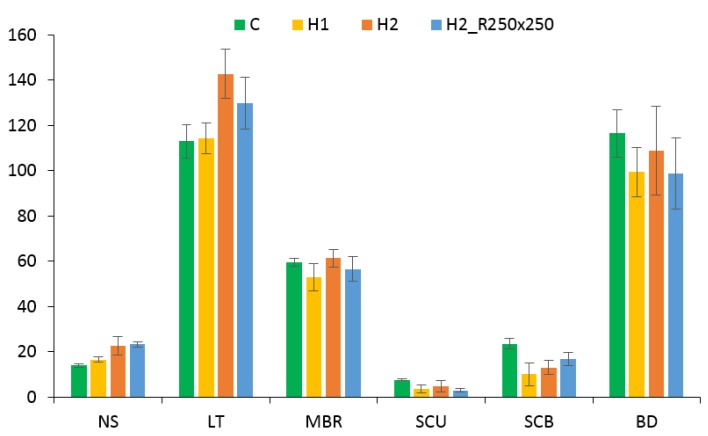
Morphology traits of plants grown in different experiment variants; NS = number of stomata, LT = leaf thickness (µm), MBR = main bundle radius (µm), SCU = sclerenchyma cells–top, SCB = sclerenchyma cells – bottom, BD = bundles distance (µm).

Closest phenotype to C could be observed in plants grown in H2 soil, where leaves were green with light purple edges (Figure 6c), they lack some sclerenchyma, compared to C, but resemble developmental stage of C plant (Figure 7 c,d and Figure 8). Its stomata were organized in two rows or one and two rows (Figure 7g,h). In H1 plants, alterations of xylem could be seen (Figure 7b, red arrow), according to shape and number of bundles, and seems to be underdeveloped. Size and number of motor cells was much lower than in C plants and plants cultivated in soil H2. Overall color of the leaf was faint with dark purple edges (Figure 6b), which is typical for undernourished leaves. Similar features were observed in leaves of plants treated by high doses of Regoplant (H2_R250 × 250). Compared to H1, H2_R250 × 250 had much more stomata and thicker leaves (Figure 8).

The measurement of anatomical traits (Figure 8) showed changes in certain plants. It could be concluded that stomatal density and leaf thickness increased in plants grown in soil type H2, on the other hand size of sclerenchyma regions above and under big bundles in H2 and H1 plants decreased along with distance between small vascular bundles. Main vein size remained almost unchanged.

## 4. Discussion

### 4.1. Nutrition Stress

Considering, that C plants were incubated under the same conditions as H1 and H2 plants and the only difference was the soil, the differences in physiological and morphological parameters observed among plants can be explained by different soil properties. According to Strašil et al. [52], the appropriate soil pH for growing miscanthus is 5.5–6.5. This is optimal for most plants because slightly acidic soil pH ensures good availability of most micronutrients. In our case, both types of poor soil were in optimal pH range, therefore changes in plant fitness had to be caused by another reason.

It is known that miscanthus nutrient requirements are low [53]. However, they are not zero and yields in marginal soils are significantly lower compared to good quality soils [18]. Contradictory effects of fertilization on miscanthus production were published. In the review Cadoux et al. [53] summarized that most studies reporting no observed effect of fertilization on the miscanthus production were established at soils with high nutrients and the monitoring was short-term only. These studies are thus not very relevant to standard long-term production of miscanthus biomass. According to recently published papers, if fertilization is applied at low quality marginal soils, the effect is significant and it becomes more relevant after the third year of cultivation [54,55]. Additionally Pogrzeba et al. [9] determined a significant effect of NPK (nitrogen, phosphorus, potassium) fertilization on photosynthesis rate, transpiration and stomatal conductance in Mxg grown in metals-contaminated agricultural soil. Obviously, there is some relationship between miscanthus vitality and nutrition supply. Effect of nutrition deficit on miscanthus physiology was recently studied by Da Costa et al. [56]. They did not find a significant effect on quantum yield efficiency (*Fv*/*Fm*) and stomatal resistance but chlorophyll content was significantly reduced in nutrient deficit soil. Additionally, they observed increased percentage of “yellow pixels” at visual spectrum images of plants indicating leaf senescence and stress response symptoms.

### 4.2. Physiological Status and Changes in Leaf Fluorescence

Measurement of fluorescence in Stimpo and Regoplant-treated plants resulted in different shapes of induction curves. Results differed also for different types of soil used. Surprisingly, higher concentration of PGR did not “improve” the shape of the curve and the situation was often opposite, than expected: the most evident example is in OJIP curve of plants treated by highest concentration of Regoplant (H2_R205 × 250), which is the lowest of all presented. Typical for various types of stress is presence of so called “K-band” (between 200–300 µs). Appearance of this peak is usually connected with disruption of oxygen evolving complex (OEC) in PSII [57]. K-band was not visible in any of our plants, treated or non-treated.

Double normalization of O-K part of the curve, revealed presence of so called “L-band” in H1 Stimpo treated plants. It is possible, that Stimpo influences excitation energy transfer between PSII units, expressed by L-band [58]. On the other hand, effect of Regoplant can be identified from Figure 3 by decrease of the amplitude of the OJIP curve between “I and P” phase (30–300ms). In R250 × 250, both, H1 and H2 plants, we observe very low difference between I and P phase, suggesting negative effect of Regoplant on PSI. 

Additionally, 24 physiological parameters were evaluated in order to detect the possible positive effect of PGRs for stress reduction in the system. As seen in Figure 2 and Figure 4, one parameter remained almost unchanged (*Vj*) in treated and non-treated plants, some parameters display change in treated or only in non-treated plants like *Fo*/*Fm*, *Fv*/*Fm*, *Fv*/*Fo*, *ABS*/*CSo*, *DIo*/*CSm*, some parameters vary between Stimpo and Regoplant treatment (*DIo*/*CSo*, *TRo*/*Cso*, *ETo*/*RC*, *REo*/*RC*, *PIabs*, *TRo*/*CSm*) and even within one type of treatment: for example for *DIo*/*RC* (Regoplant) and *DIo*/*RC* and *ABS*/*CSm* (Stimpo) changes between concentrations of applied PGR can be found, implying that these parameters can be used as highly sensitive markers to minor changes in plant photochemistry. 

Unfortunately, not all available instruments provide this type of measurement. Therefore the common *F_v_*/*F_m_* parameter [21,28] was also included in this study for comparison. It should be noted that some authors consider *F_v_*/*F_m_* ratio as parameter with low sensitivity [49]. In our case also the JIP-test was not able to show convincing difference between Stimpo and Regoplant treated individuals in H1 soil. Therefore we decided to perform double normalization of transient 50 µs–300 µs (W_OK_), which is the most sensitive part of the curve [49]. We can conclude that this type of analysis, can visualize finer differences between variants as obvious from Figure 5, where we can clearly distinguish between Stimpo and Regoplant treated plants in H1.

Chlorophyll a induction curves were used to visualize fluorescence intensities in different types of soils as well. Plants grown in H1 and H2 display much lower curves than plants grown in compost (C plants). Changes in shape and slope of curves are typical for abiotic stress in plants [25,36,41]. Lower *Fo* and *Fm* values have been observed as result of different types of stress, lower *Fv*/*Fm* (in our case pre-dawn *Fv*/*Fm*), can therefore indicate substantial down-regulation of photosystems (PSII) due to photoinhibition [27]. It was documented before that deficiency of nitrogen, potassium, sulfur, phosphorus, magnesium and calcium can lead to disruption of photosynthetic apparatus. Some authors confirm, in accordance with our research, that low nutrition results in decrease of photochemical activity and change of fluorescence parameters [59].

Absorption in H2 and H1 plants is much lower than in C plants, on the other hand, but dissipation of energy per reaction center does not differ too much. That implies that C plants might have larger light harvesting complexes (LHC), compared to H1 and H2 plants. That is in accordance with apparently better shape of C plants.

Despite the fact, that mechanism of Stimpo and Regoplant “act” can differ, application of each PGR resulted in different effect. Application of Stimpo increased capture (*TRo*/*RC*), dissipation (*DIo*/*RC*) and transport of electrons (*ETo*, *REo*/*RC*) in H1 plants only. Regoplant increased same parameters (*DIo*, *TRo*, *REo*/*RC*) only in H2 treated plants, electron transport flux was not affected by Regoplant application. Regardless of stimulant used, soil type seems to be affecting these parameters as well. Increase of parameters like *TRo*/*RC* and *REo*/*RC* was observed in low-nutrition stressed plants [60]. Dissipation of absorbed energy as heat can be sign of downregulation of photochemical activity as result of ongoing stress. H1 and H2 soil do not have very different composition, but they are both nutritionally very poor (compared to C). Combined with effect of Stimpo or Regoplant, specific reactions can be observed in terms of fluorescence.

Nevertheless, fluorescence-based parameters are not stress-specific. Thus, the investigation of plant leaf structure by independent, but invasive, method was performed.

### 4.3. Changes in Leaf Anatomy

The various changes in leaf anatomy were observed starting with increased number of stomata in H1 and H2 plants compared to C. It is known that an increase in stomatal density is often connected to influence of drought and environmental factors [61]. In our case, plants were watered regularly, so we consider the effect of drying of the substrate negligible.

The other typical feature for H1 and H2 plants was lower amount of sclerenchyma cells in comparison with standard C plant. Moreover, H1 plants differ in sclerenchymatic tissues compared to H2 plants with slightly higher nutrients content. Similar results were observed in other studies. For example it was proved that lack of potassium in rice can lead to decreased amount of sclerenchyma [62]. Significant role of potassium for synthesis of stability tissues (like sclerenchyma) was observed for other crops as wheat [63] and oilseed rape [64].

The anatomical changes were reported after exposure to abiotic stress as well. Pitman et al. [65] observed increase in sclerenchyma cells in kleingrass subjected to water stress. Some other changes in anatomical traits were observed in plants being exposed to stress. Bilska-Kos et al. [40] applied cold treatment to young Mxg plants and after three days, leaf thickness as well as bundle sheath area increased. Bundle sheath distance varied between experimental variants. Similar to our treated plants, Makbul et al. [66] observed decrease in amount of sclerenchyma in soybean stems in drought stressed plants, compared to standard plants.

### 4.4. Effect of PGRs

Both PGRs contain various compounds and extracts which were expected to stimulate plant growth and reduce negative effect of stress conditions. As reported by Ponomarenko et al., when energy crops grew in agricultural soil, these PGRs stimulated nutrient uptake, plants grew well and used strong photosynthetic apparatus for production of large amount of biomass [20]. However, as it was observed in the current study when Mxg grew in nutritionally poor soil, stimulation by PGRs did not result in better physiological state of plants. The same effect was observed for biomass yield in our previous study [19]. With exception of Stimpo applied only to rhizomes, in case of H2 soil which is little more favorable in nutrients content, the situation was even worsened by PGR application. 

The probable reason of that negative effect was the stimulatory effect of those substances which is otherwise desirable. The worst results were obtained for combined application of Regoplant, which contains synthetic analogue of plant auxin (1-NAA) known for its stimulatory effect on plant growth. But in this case, stimulatory effect acted contradictory as it could not be fulfilled, due to a lack of nutrients, and contributed to plant depletion. 

### 4.5. Recommendations for Miscanthus Cultivation in nutritionally Poor Soil

Here we confirmed that growing Mxg in nutritionally deficit soil negatively affects its physiological state. Since the lack of nutrients is a common problem of many marginal sites, it is important to look for a way to reduce its negative effect on biomass production. As PGR application does not seem to be an effective way in this case, the efforts should be focused on soil quality improvement. It could be done by NPK fertilization (as mentioned in chapter 4.1) or by application of various soil amendments. For example Kharytonov et al. [67] described positive effect of ash and sludge on Mxg biometric parameters and productivity in mining soils. It can be presumed based on biomass results [19] that after soil improvement also PGR application can bring additional positive effect.

It was found by da Costa et al. [56] that Mxg is less tolerant to nutrient deficiency and combination of nutrition and drought stress than its parental species *M. sacchariflorus* and particularly *M. sinensis*. On the other hand, these two species are, contrary to Mxg, seed-based so it is necessary to consider the possibility of their invasive spread in the environment. Thus, any currently available variant is not ideal. For the future, a very promising solution is the development of new hybrids which are more stress tolerant and provide higher biomass yields in marginal soils compared to currently used genotypes [17,68].

## 5. Conclusions

Application of two PGRs Stimpo and Regoplant was tested for reduction of stress level of energy crop *Miscanthus x giganteus* grown in nutritionally poor post-military soil. It was verified that measurement of plant leaf fluorescence can serve as a powerful tool to detect plant stress *in vivo*. While determination of common parameters *Fv*/*Fm* and P.I. is considered a suitable method for the identification of major plant stress, here we demonstrate that finer changes in plant fitness can be hidden from these two parameters. Nevertheless, we observed that the minor alterations can be revealed using other indexes. Dissipation and trapping flux per reaction center (*DIo*/*RC* and *TRo*/*RC*) and also electron transport to PSI electron acceptors (*ETo*/*RC*), seem to be highly sensitive markers for detection of the minor changes in plant photochemistry. 

The substantial changes in leaf morphology, i.e., increased number of stomata and lower amount of sclerenchyma cells in plants grown in poor soils were found. Since it was established that application of PGRs Stimpo and Regoplant did not reduce the stress level of Mxg, the direct improvement of soil shall be considered for stress reduction.

## Figures and Tables

**Table 1:** Chemical and microbial parameters determined in soils used in experiment.

Parameter	H1	H2	C
pH (H_2_O)	5.89	6.30	7.58
Available P [mg/kg]	24	49	1227
Available K [mg/kg]	44	57	3620
Available Ca [mg/kg]	74	365	12,917
Available Mg [mg/kg]	19	40	1757
Ntot [%]	0.02	0.02	1.52
S [%]	0.33	0.19	1.21
Humus [%]	1.32	0.77	33.11
PLFAtot [mg/kg]	3.11 ± 0.69	2.31 ± 0.62	22.30 ± 3.27
G+/G− PLFA	0.49 ± 0.12	0.31 ± 0.04	0.84 ± 0.04
F/B PLFA	0.14 ± 0.03	0.18 ± 0.13	0.08 ± 0.01
Respiration [nmol/min/g]	2.16 ± 0.51	0.86 ± 0.46	22.13 ± 4.97

H1, H2 = Hradčany 1 and Hradčany 2 soils (poor in nutrients), C = Compost (control, rich in nutrients). PLFAtot: Sum of concentration of all fatty acids methylesters (FAMEs) C10–C20, G+/G− phospholipid fatty acids (PLFA): Ratio of sum of indicator FAMEs for Gram-positive and Gram-negative bacteria, F/B PLFA-ratio of sum of indicator FAMEs for fungi and bacteria.

**Table 2:** Composition of used plant growth regulators (PGRs) (adapted from Nebeská et al. [19] and http://www.agrobiotech.com.ua), components which are specific for sole PGR are in bold.

PGR Title	Stimpo	Regoplant
Standard	TU U 20.2-31168762-005:2012	TU U 20.2-31168762-006:2012
Description	Balanced composition of biologically active compounds: analogues of phytohormones, amino acids, fatty acids, oligosaccharides, microelements, and bioprotective compounds	Balanced composition of biologically active compounds: analogues of phytohormones, amino acids, fatty acids, oligosaccharides, chitosan, microelements, and bioprotective compounds
Composition	Emistim C: *Cylindrocarpon obtusiusculum*-(auxin phytohormones, cytokinin nature, saturated and unsaturated fatty acids, amino acids), carbohydrates, ion biogenic microelements	Emistim C: *Cylindrocarpon obtusiusculum* - (auxin phytohormones, cytokinin nature, saturated and unsaturated fatty acids, amino acids), carbohydrates, ion biogenic microelements
	Microbial pesticide “Actofit, 0.2% к.e.”: Natural complex Aversectin C, a product of vital activity of actinobacterium *Streptomyces avermytilis*	Microbial pesticide “Actofit, 0.2% к.e.”: Natural complex Aversectin C, a product of vital activity of actinobacterium *Streptomyces avermytilis*
	Microelements: Acid boron, Copper sulfuric acid (II) 5-water, ammonium, molybdenum acid, Manganese (II) chloride 4-water, Potassium iodide	“Reakom”: Composition of biogenic microelements (microfertilizer universal on the basis of micronutrient complexonates)
	K, **Na**, Fe, Zn, Mn, Cu, Mg, Ca, Co	K, Fe, Zn, Mn, Cu, Mg, Ca, **S**, **Mo**, **B**, **N**
	Ethanol	**Brilliant green**
	Purified water	**Potassium salt of 1-naphthylacetic acid C_12_H_9_KO_2_**
		Ethanol
		Purified water

**Table 3:** Experiment variants.

Variant Label	Soil	PGR	PGR Concentration [mL/10 L]		Number of Pots
			**Soaking**	**Spraying**	
C	Compost	-	-	-	3
H1	Hradcany 1	Water	0	-	3
H2	Hradcany 2	Water	0	-	3
H1_S50x0	Hradcany 1	Stimpo	50	-	3
H2_S50x0	Hradcany 2	Stimpo	50	-	3
H1_S50x50	Hradcany 1	Stimpo	50	50	3
H2_S50x50	Hradcany 2	Stimpo	50	50	3
H1_R250x0	Hradcany 1	Regoplant	250	-	2
H2_R250x0	Hradcany 2	Regoplant	250	-	2
H1_R250x250	Hradcany 1	Regoplant	250	250	2
H2_R250x250	Hradcany 2	Regoplant	250	250	2

## Supplementary Materials

The following are available online at https://www.mdpi.com/2223-7747/9/2/194/s1, Table S1: Means and standard deviations of fluorescence indexes for all PGRs concentrations in experiment; Figure S2: Boxplots of fluorescence indexes; Figure S3: Photograph of the experiment; Figure S4: Average month temperatures, precipitation and light period in Ústí nad Labem in 2017.

## Appendix A-Abbreviations

Adapted from Hansatech Instruments and [34].

*F_o_*	zero fluorescence, fluorescence level when plastoquinone electron acceptor pool (Qa) is fully oxidized; these acceptors are available to receive light energy due to dark adaptation.
*F_m_*	maximum fluorescence, fluorescence level when all electron acceptors are fully reduced, no longer available for photochemistry.
*F_v_*	variable fluorescence (F_m_ − F_o_).
*F_j_*	fluorescence intensity at 2 ms.
*F_i_*	fluorescence intensity at 30 ms.
*F_k_*	fluorescence intensity at 300 µs.
*F_v_*/*F_m_*	maximum quantum efficiency of Photosystem II; maximum quantum yield of primary photochemistry; typical value for non-stressed plant is 0.85 [69], can be decreased by biotic or abiotic stress; F_v_/F_m_ = (F_m_ − F_o_)/F_m._
*T_fm_*	time to achieve maximum fluorescence; shorter T_fm_ can indicate stress of the plant.
*M_0_*	slope at the origin of the fluorescence rise; M_0_ = 4 (F_k_ − F_o_)/(F_m_ − F_o_).
*V_j_*	relative variable fluorescence at 2 ms; V_j_ = (F_j_ − F_o_)/(F_m_ − F_o_).
*V_i_*	relative variable fluorescence at 30 ms; V_i_ = (F_i_ − F_o_)/(F_m_ − F_o_).

Relative calculated values refer to specific and phenomenological energy fluxes per reaction center (*RC*) and cross section (CS) considering thickness of the leaf and illuminated mm^2^. Phenomenological energy fluxes are displayed as energy fluxes per excited cross section at fully open reaction centers (*CS_0_*, cross section of the leaf tissue at *F_o_*), or at the stage of fully closed reaction centers (*CS_m_*-cross section of the leaf at *F_m_*)

*TR_0_*/*RC*	trapping at time zero per RC; TR_0_/RC = M_0_/V_j._
*DI_0_*/*RC*	dissipation at time zero, per RC; DI_0_/RC = (ABS/RC) − (TR_0_/ABS) = M_0_ (1/V_j_) [1/(F_o_/F_m_)].
*ET_0_*/*RC*	electron transport at time zero per RC; ET_0_/RC = (M_0_/V_j_) (1 − V_j_).
*RE_0_*/*RC*	electron flux leading to the reduction of the PS I end acceptor. RE_0_/RC = M_0_ (1/V_j_) (1 − V_i_).
*ABS*/*CS*_0_	absorption at time zero per CS; ABS/CS_0_ = F_o._
*TR_0_*/*CS_0_*	trapping at time zero per CS; TR_0_/CS_0_ = (TR_0_/ABS) (ABS/CS_0_) = F_o_ [1 − (F_o_/F_m_)].
*DI_0_*/*CS_0_*	dissipation at time zero per CS; DI_0_/CS_0_ = (ABS/CS_0_) − (TR_0_/CS_0_) = F_o_ − {F_o_ [1 − (F_o_/F_m_)]}.
*ET_o_*/*CS_0_*	electron transport at time zero per CS; ET_0_/CS_0_ = [1 − (F_o_/F_m_)] (1 − V_j_) F_o._
*RE_0_*/*CS_0_*	The flux of electrons from QA- to final PSI acceptors per cross section of PSII at time zero; RE_0_/CS_0_ = [1 − (F_0_/F_m_)] (1 − V_j_) [(1 − V_i_)/(1 − V_j_)] F_o._
*ABS*/*CS_m_*	absorption at maximum time per CS; ABS/CS_m_ = F_m._
*TR_0_*/*CS_m_*	trapping at maximum time per CS; TR_0_/CS_m_ = (TR_0_/ABS) (ABS/CS_m_) = F_m_ [1 − (F_o_/F_m_)].
*DI_0_*/*CS_m_*	dissipation at maximum time per CS; DI_0_/CS_m_ = (ABS/CS_m_) − (TR_0_/CS_m_) = F_m_ − {F_m_ [1 − (F_o_/F_m_)]}.
*ET_o_*/*CS_m_*	electron transport at maximum time per CS; ET_0_/CS_m_ = [1 − (F_o_/F_m_)] (1 − V_j_) F_m._
*RE_0_*/*CS_m_*	The flux of electrons from QA- to final PSI acceptors per cross section of PSII at maximum time; RE_0_/CS_0_ = [1 − (F_0_/F_m_)] (1 − V_j_) [(1 − V_i_)/(1 − V_j_)] F_m._
*P.I.*	performance index, vitality index; PI abs = {[1 − (F_o_/F_m_)]/(M_0_/V_j_)} [(F_m_ − F_o_)/F_o_] [(1 − V_j_)/V_j_].
