# Supplementary material for: Physiological Response of Miscanthus x giganteus to Plant Growth Regulators in Nutritionally Poor Soil
Source: Plants (Basel). 2020 Feb 5;9(2):194. doi: 10.3390/plants9020194 (PMC7076640; doi:10.3390/plants9020194)

Figure S2. Boxplots of fluorescence indexes

a) Fo

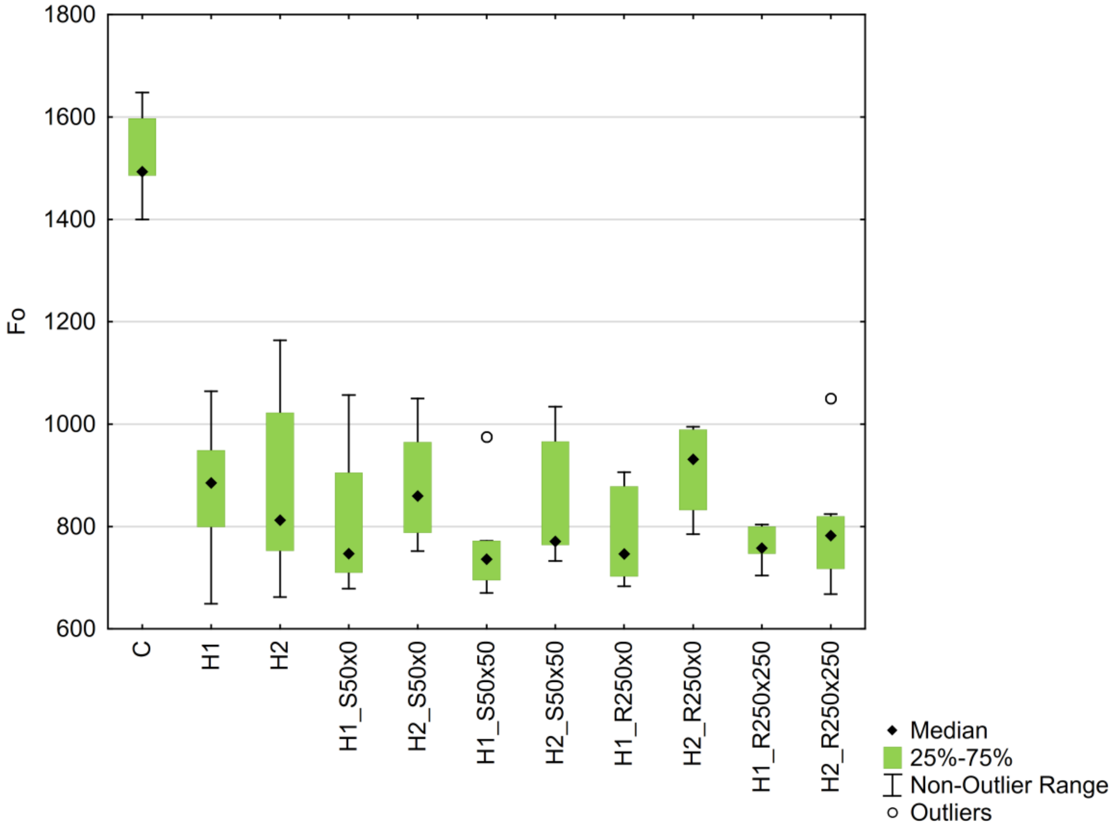

b) Fm

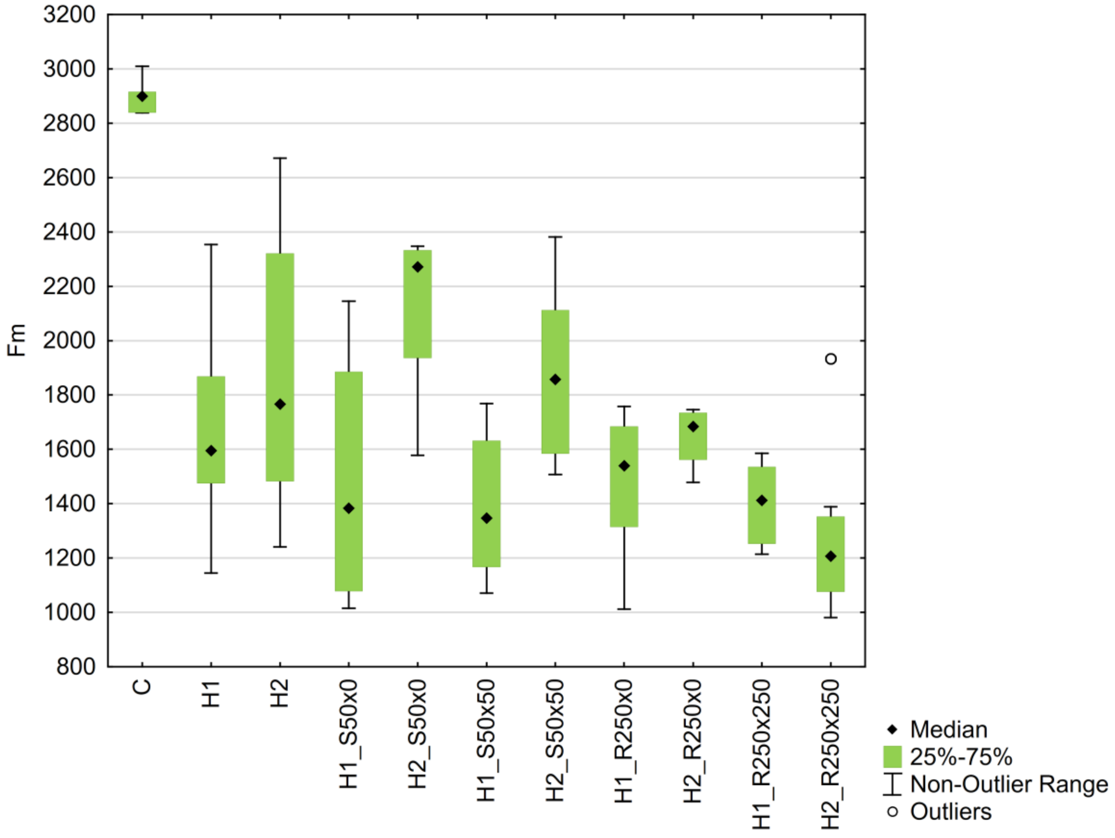

c) Fv

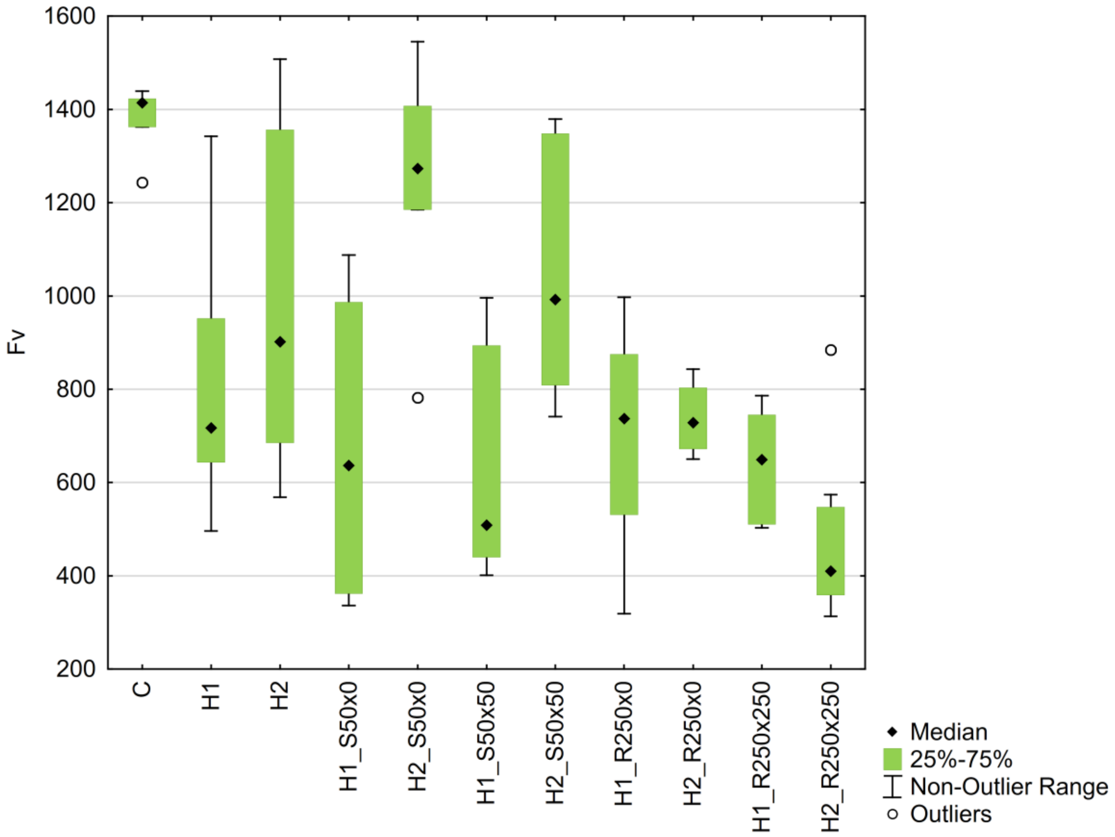
1600
1400
1200
1000
800
600
400
200
Fv
C
H1
H2
H1_S50x0
H2_S50x0
H1_S50x50
H2_S50x50
H1_R250x0
H2_R250x0
H1_R250x250
H2_R250x250
Median
25%-75%
Non-Outlier Range
Outliers

d) Fv/Fo

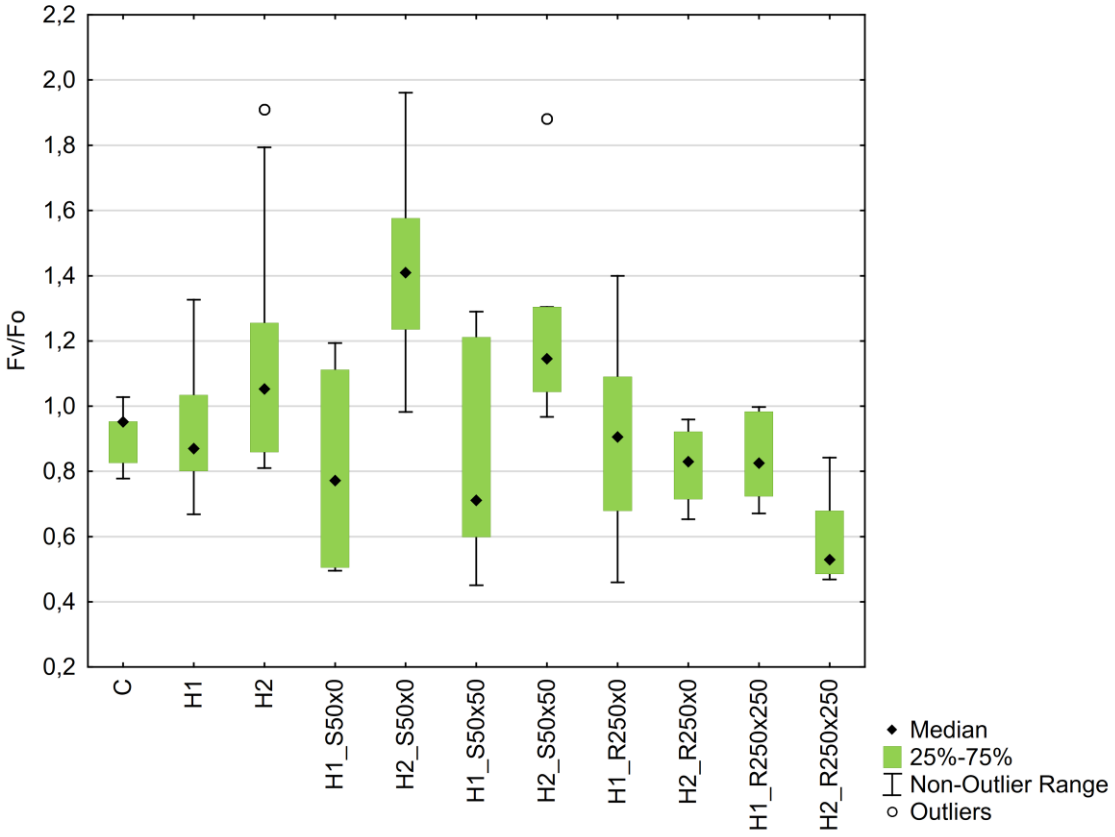
2,2
2,0
1,8
1,6
1,4
1,2
1,0
0,8
0,6
0,4
0,2
Fv/Fo
C
H1
H2
H1_S50x0
H2_S50x0
H1_S50x50
H2_S50x50
H1_R250x0
H2_R250x0
H1_R250x250
H2_R250x250
Median
25%-75%
Non-Outlier Range
Outliers

e) Vj

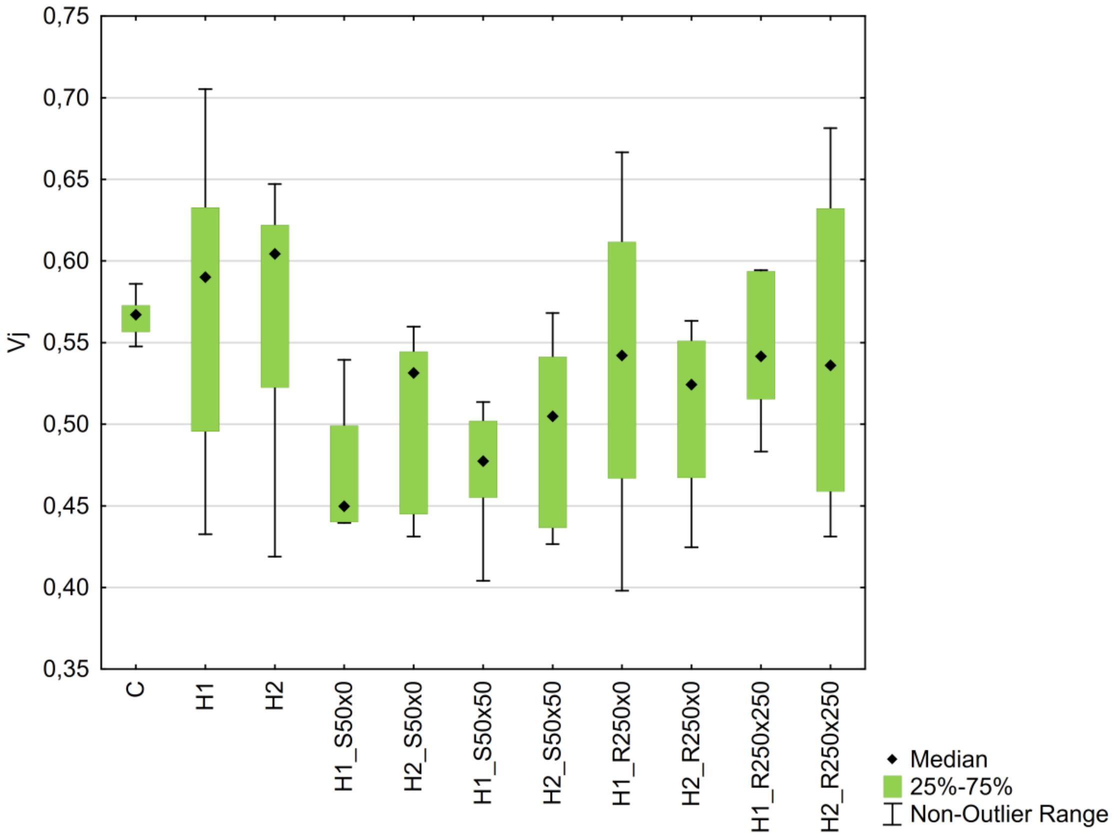

0,75
0,70
0,65
0,60
0,55
0,50
0,45
0,40
0,35
Vj
C
H1
H2
H1_S50x0
H2_S50x0
H1_S50x50
H2_S50x50
H1_R250x0
H2_R250x0
H1_R250x250
H2_R250x250
Median
25%-75%
Non-Outlier Range

f) Vi

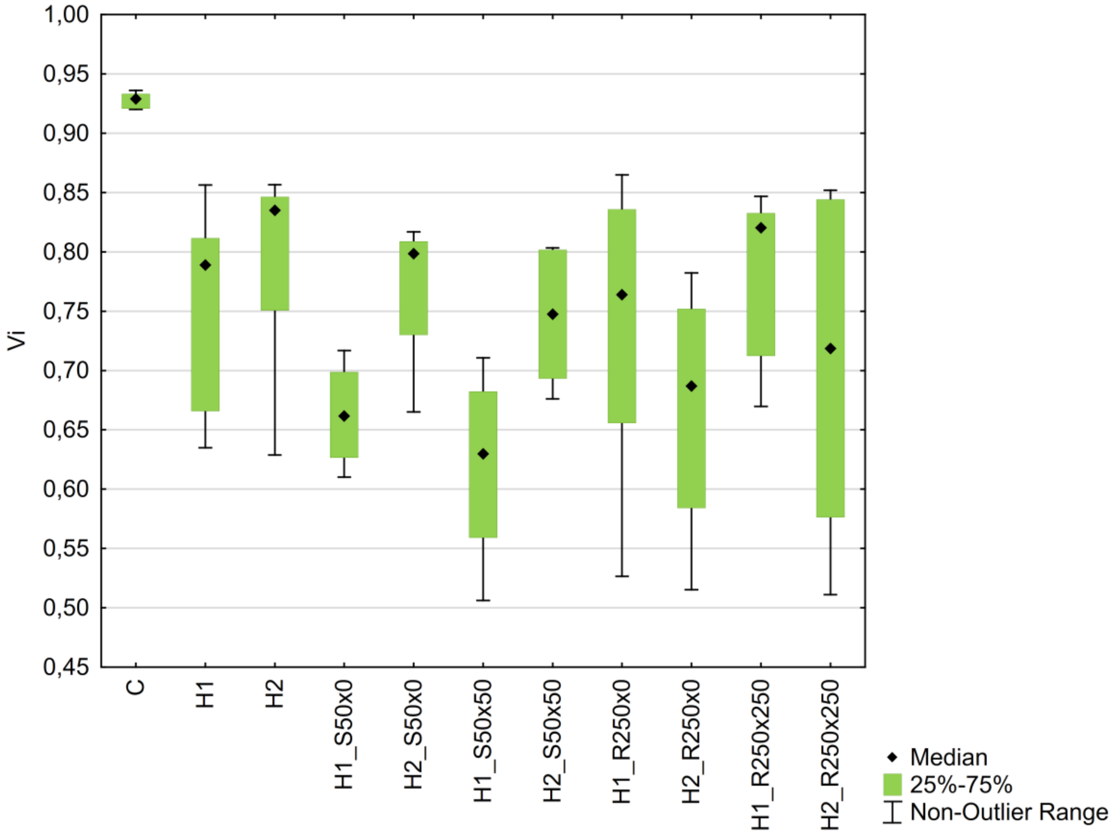

1,00
0,95
0,90
0,85
0,80
0,75
0,70
0,65
0,60
0,55
0,50
0,45
Vi
C
H1
H2
H1_S50x0
H2_S50x0
H1_S50x50
H2_S50x50
H1_R250x0
H2_R250x0
H1_R250x250
H2_R250x250
Median
25%-75%
Non-Outlier Range

g) ABS/CSm

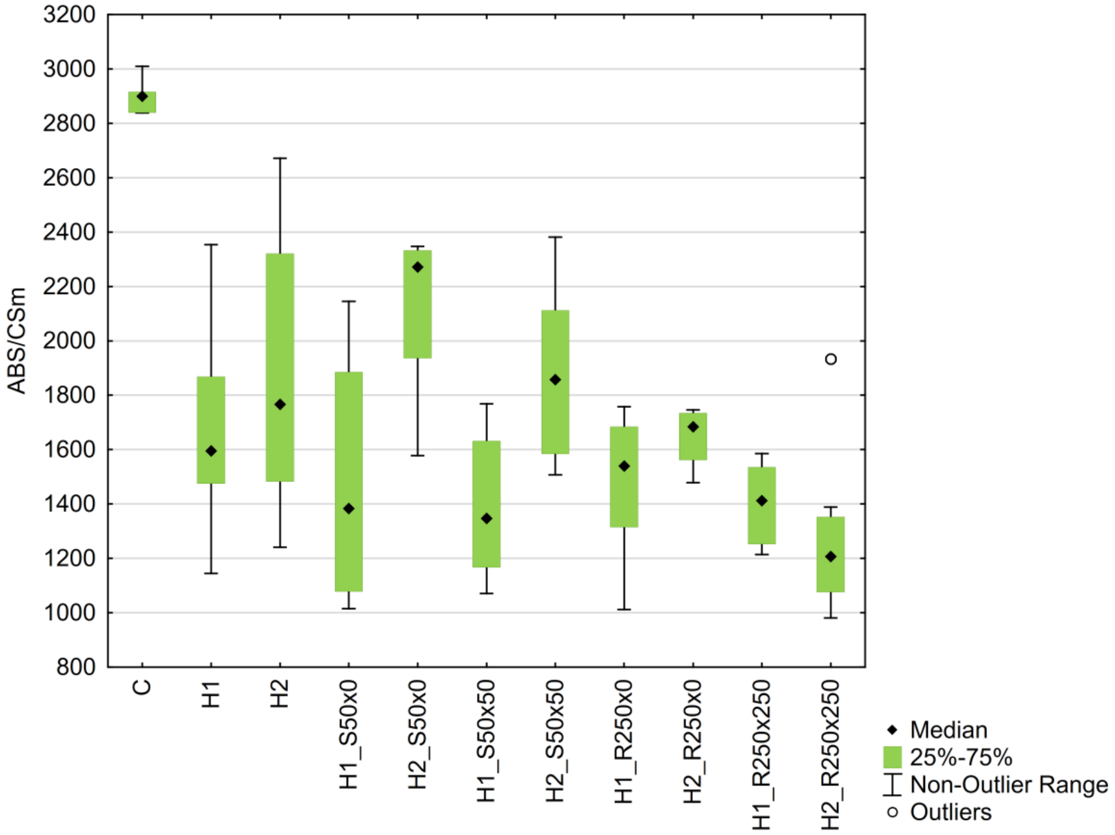

h) TRo/RC

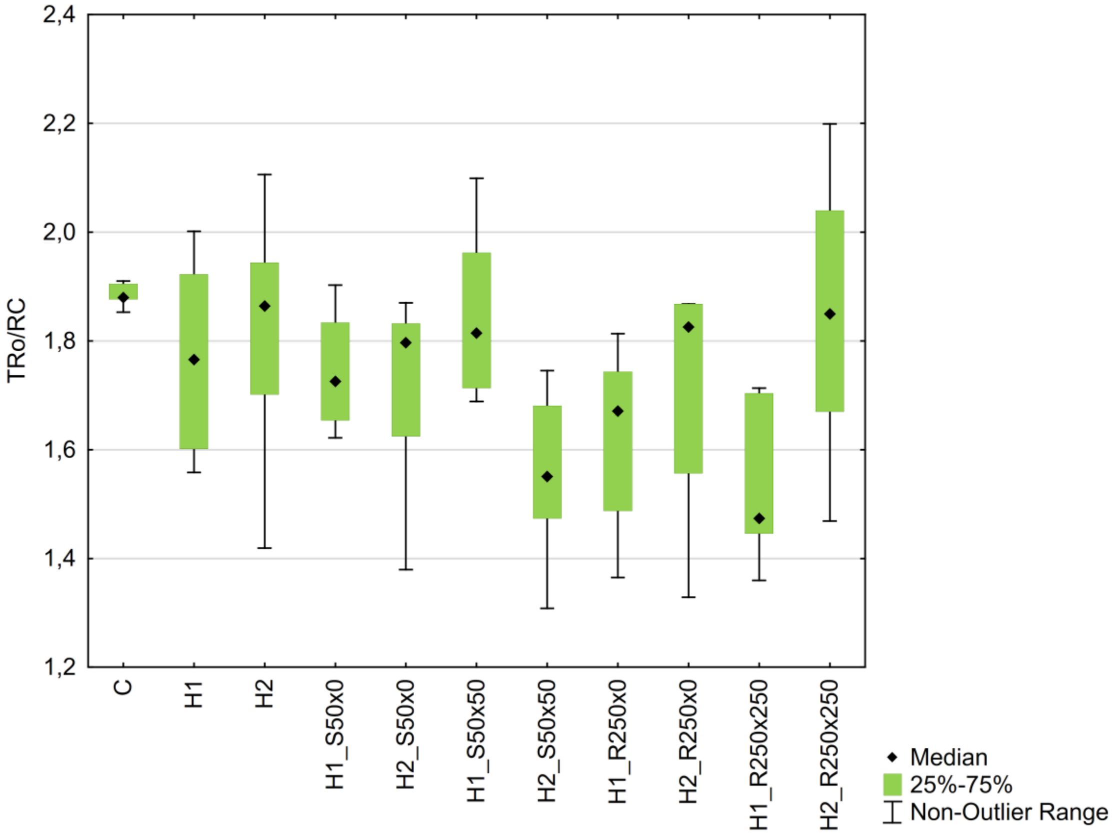

i) DIo/RC

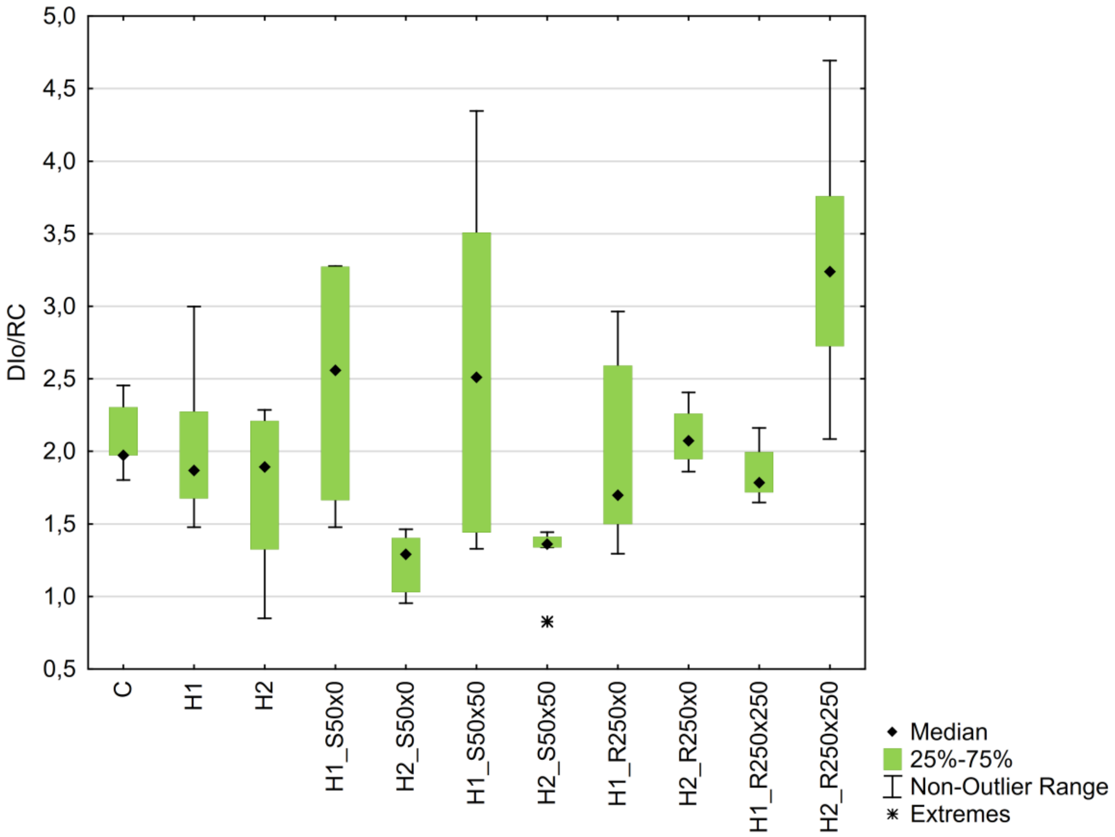

j) ABS/CSo

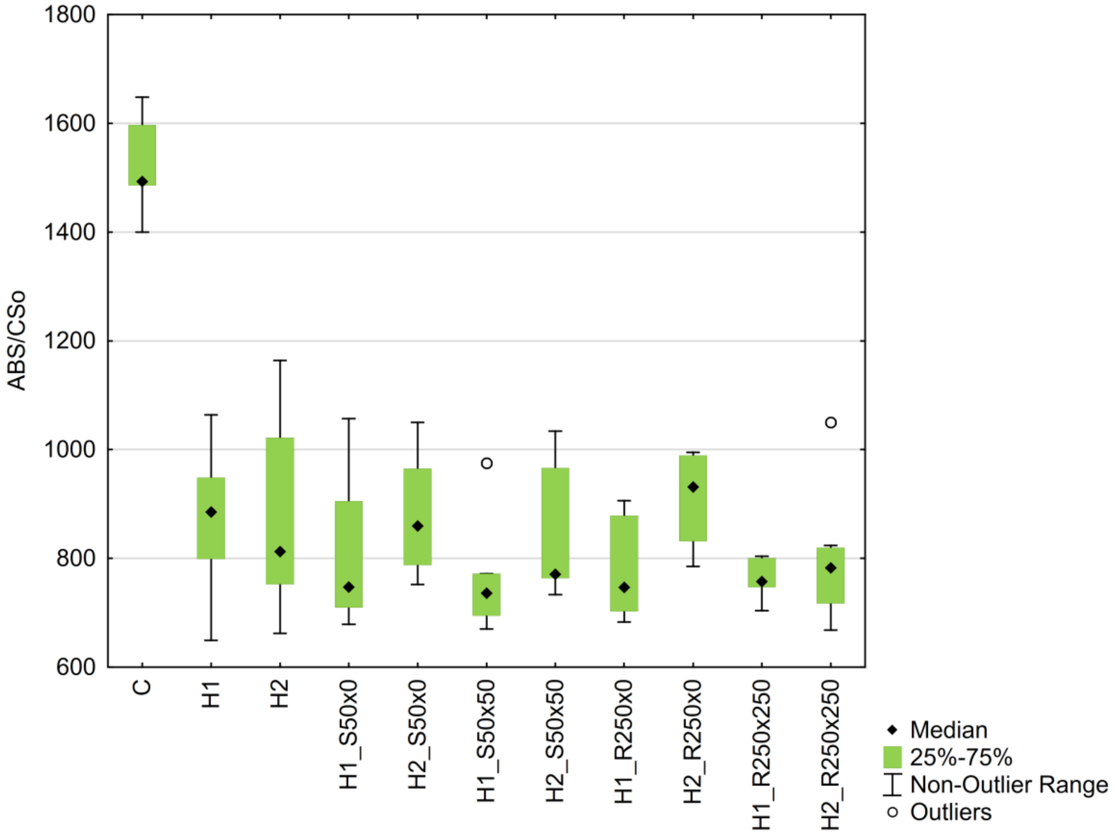

k) ETo/RC

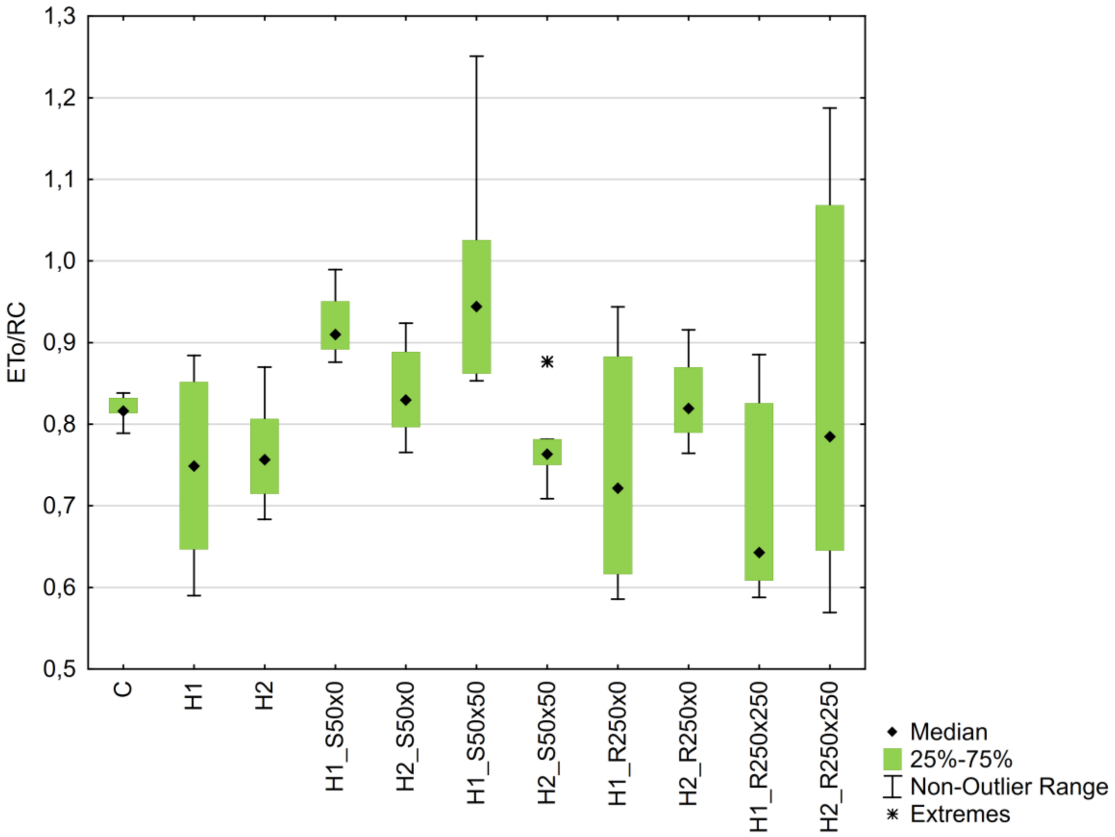

l) DIo/CSm

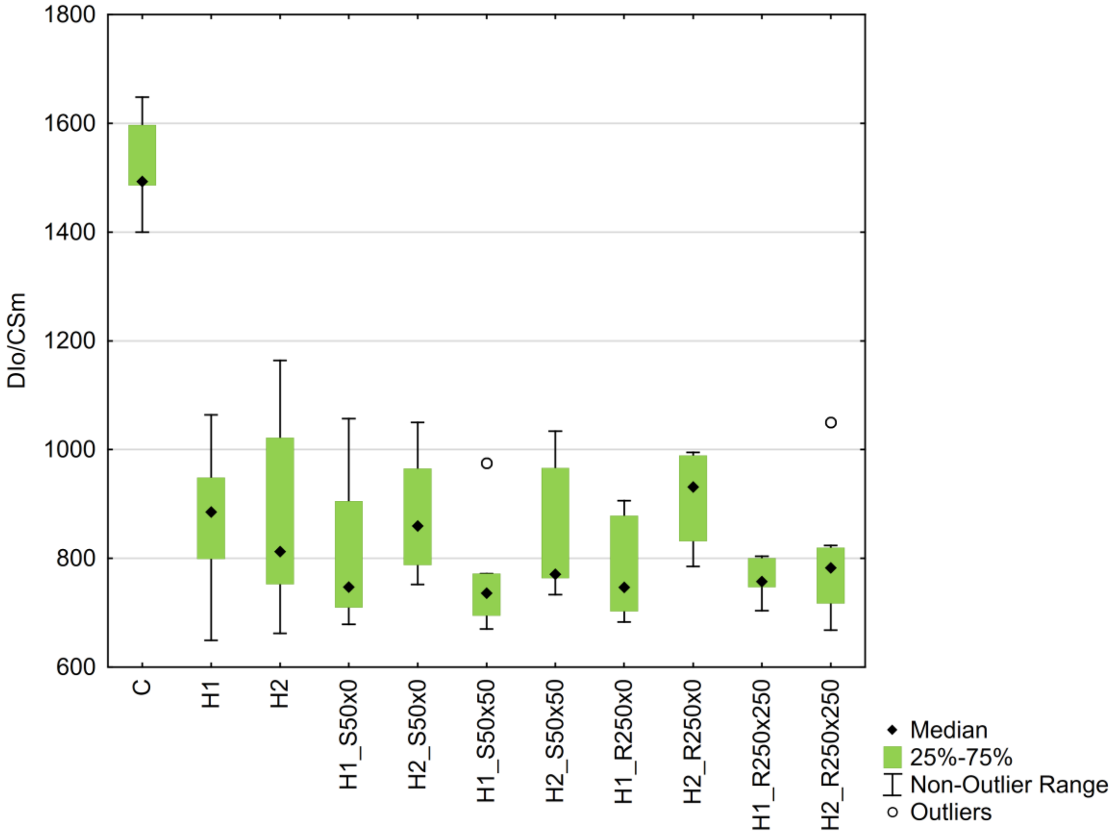

m) TRo/CSm

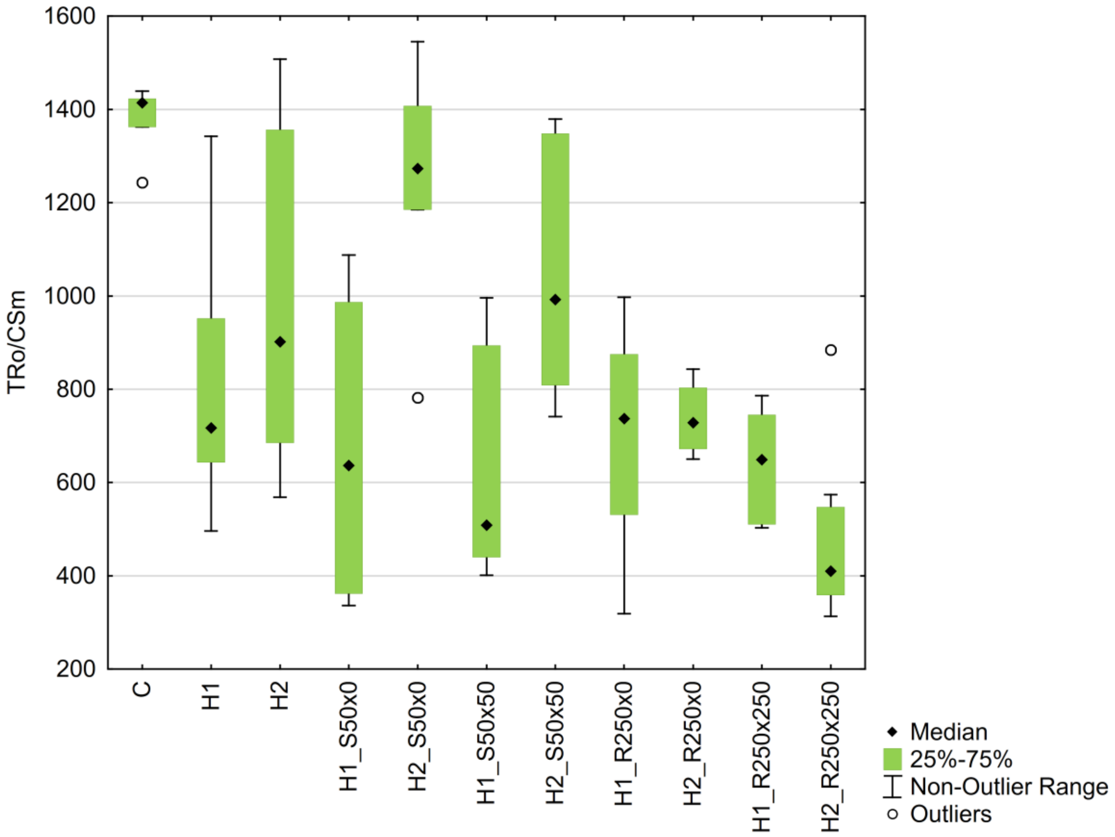

n) Tfm

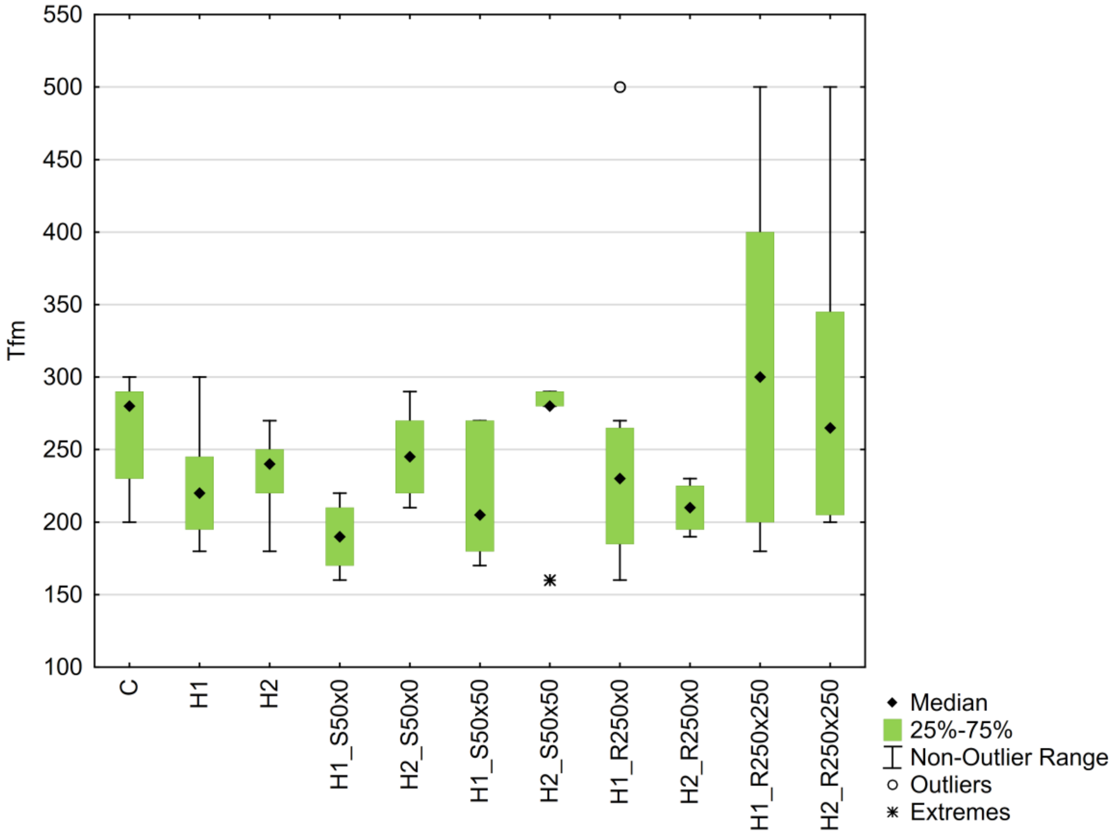

o) ETo/CSm

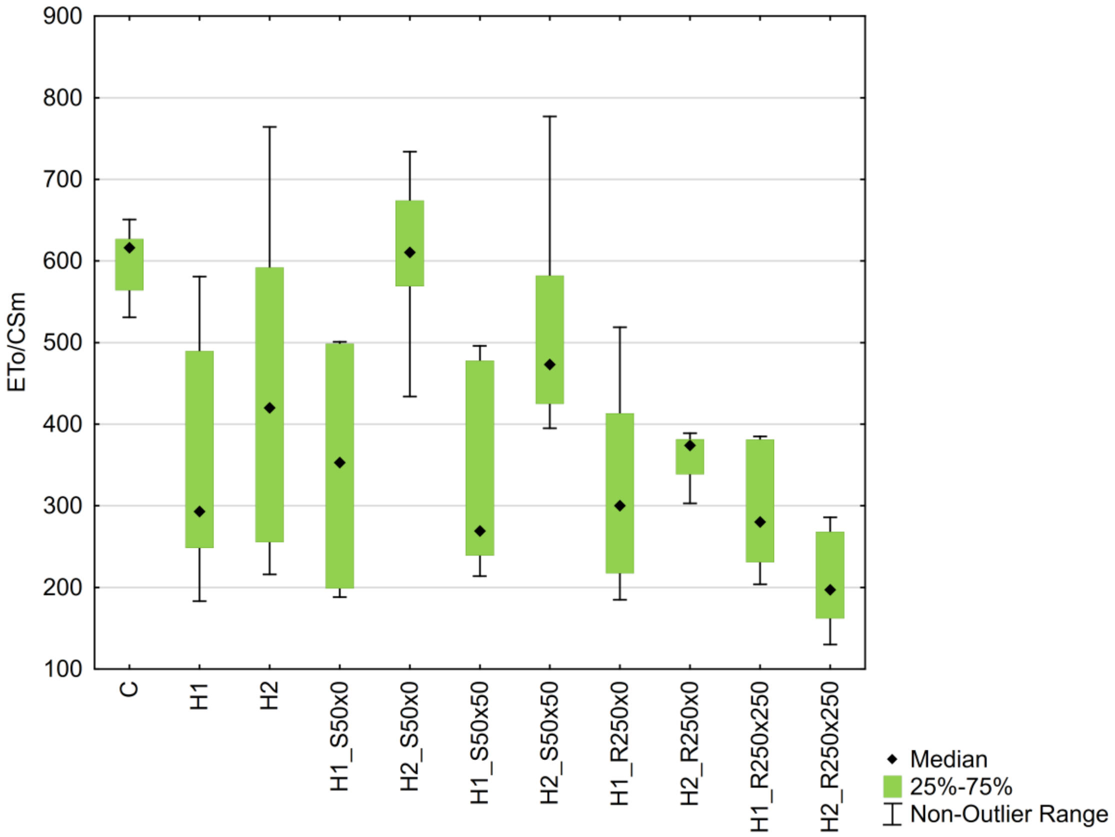

p) Fo/Fm

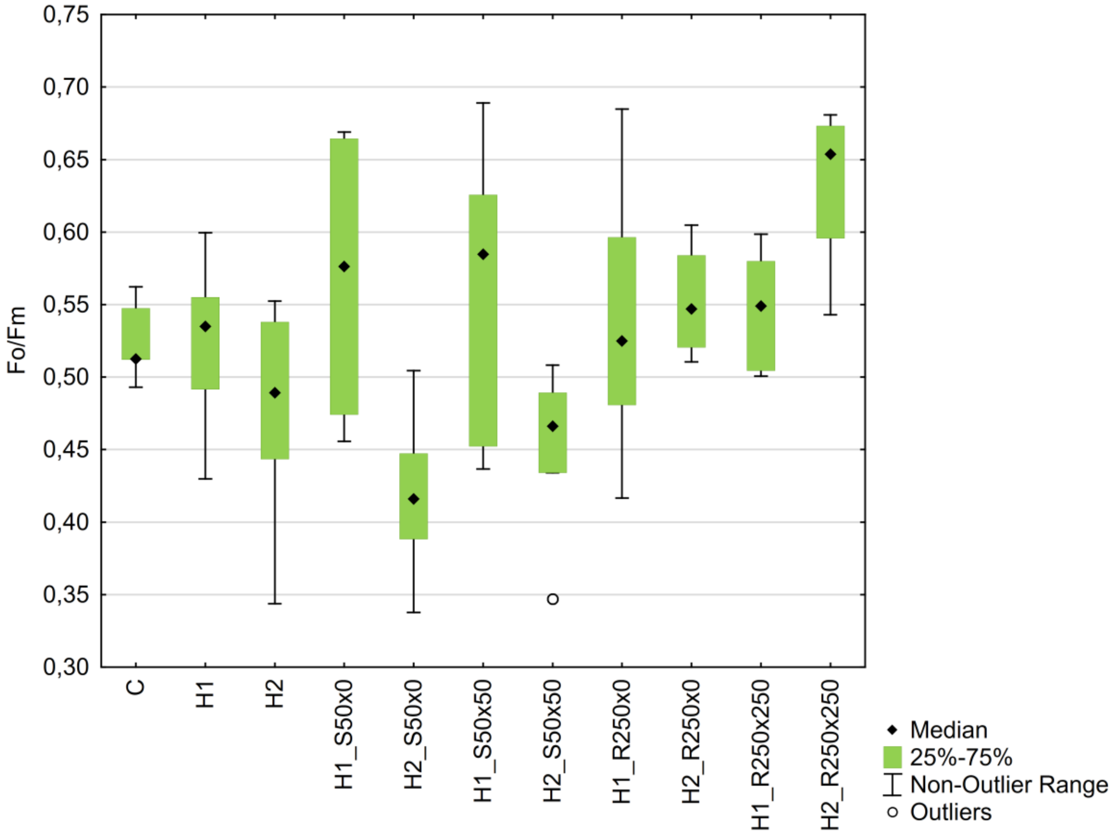

q) Fv/Fm

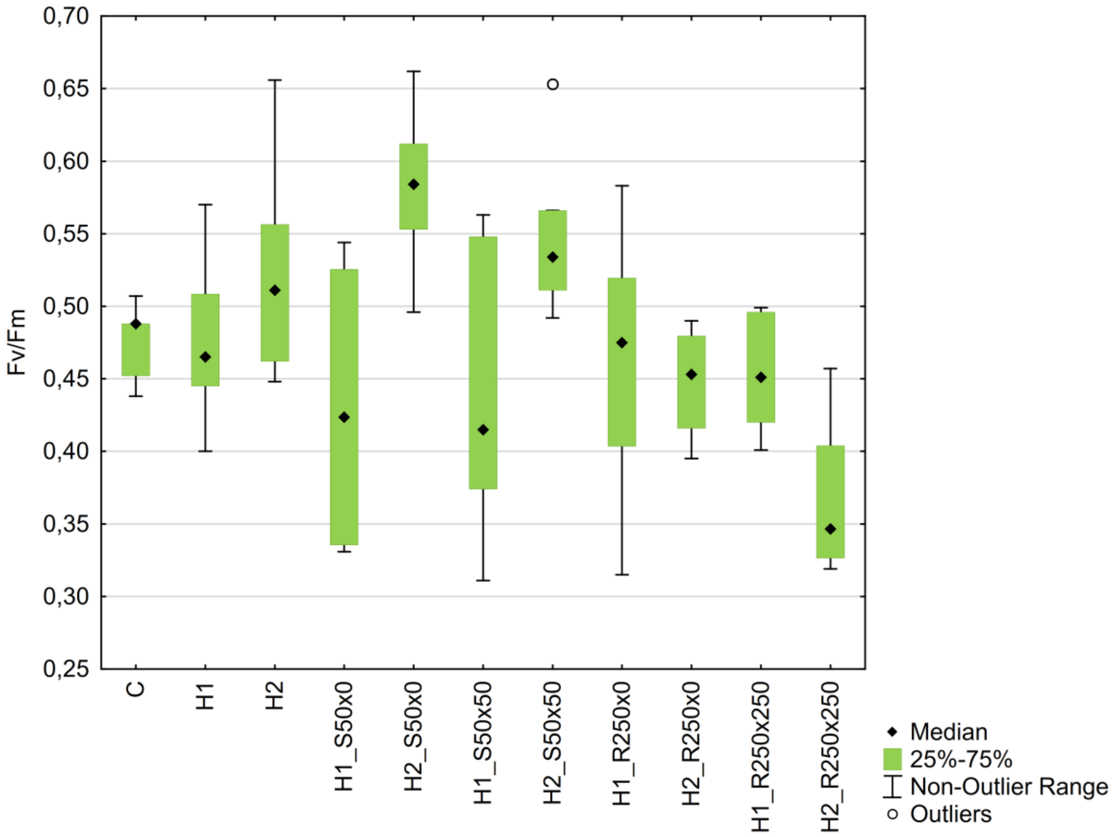

r) DIo/CSo

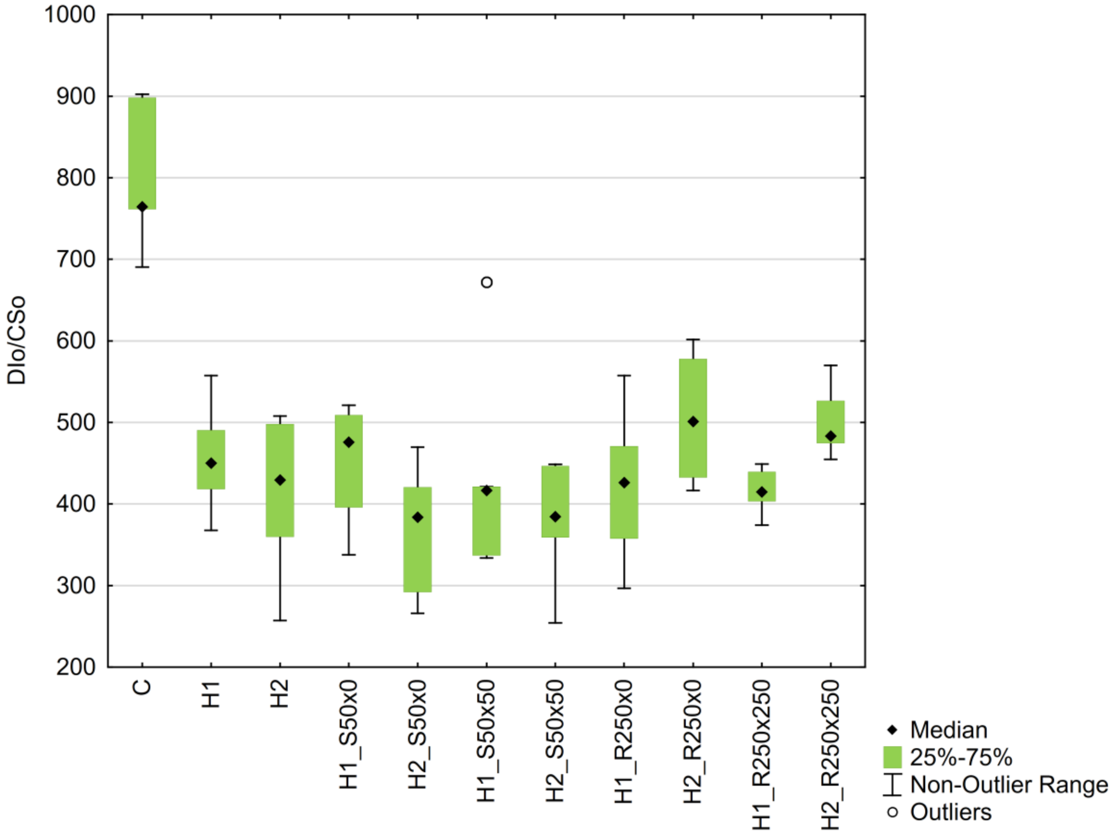

s) TRo/CSo

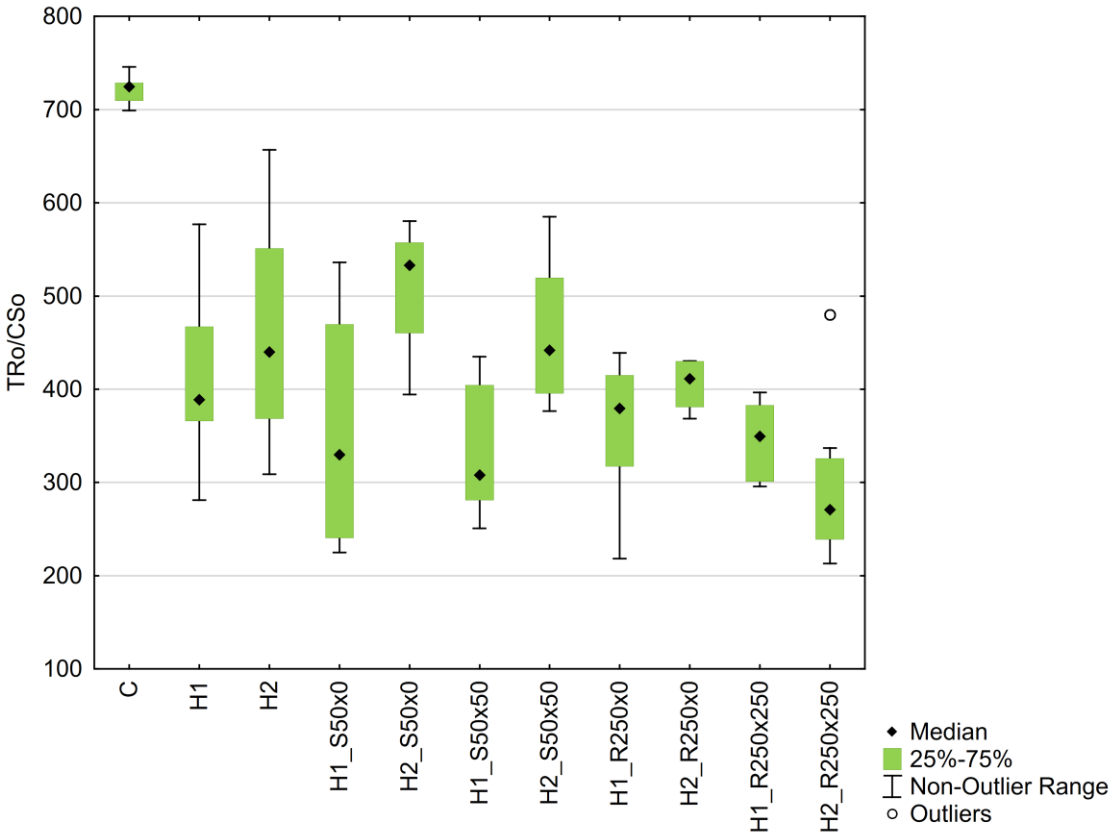

t) ETo/CSo

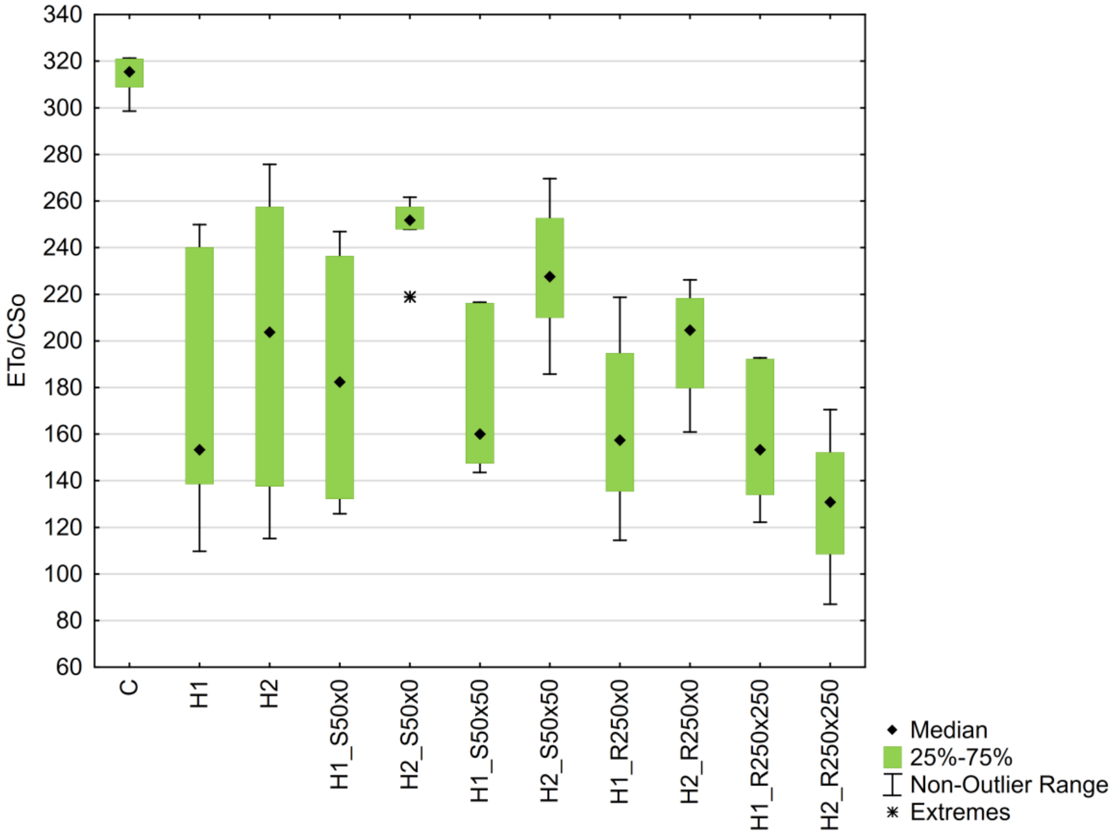

u) REo/CSo

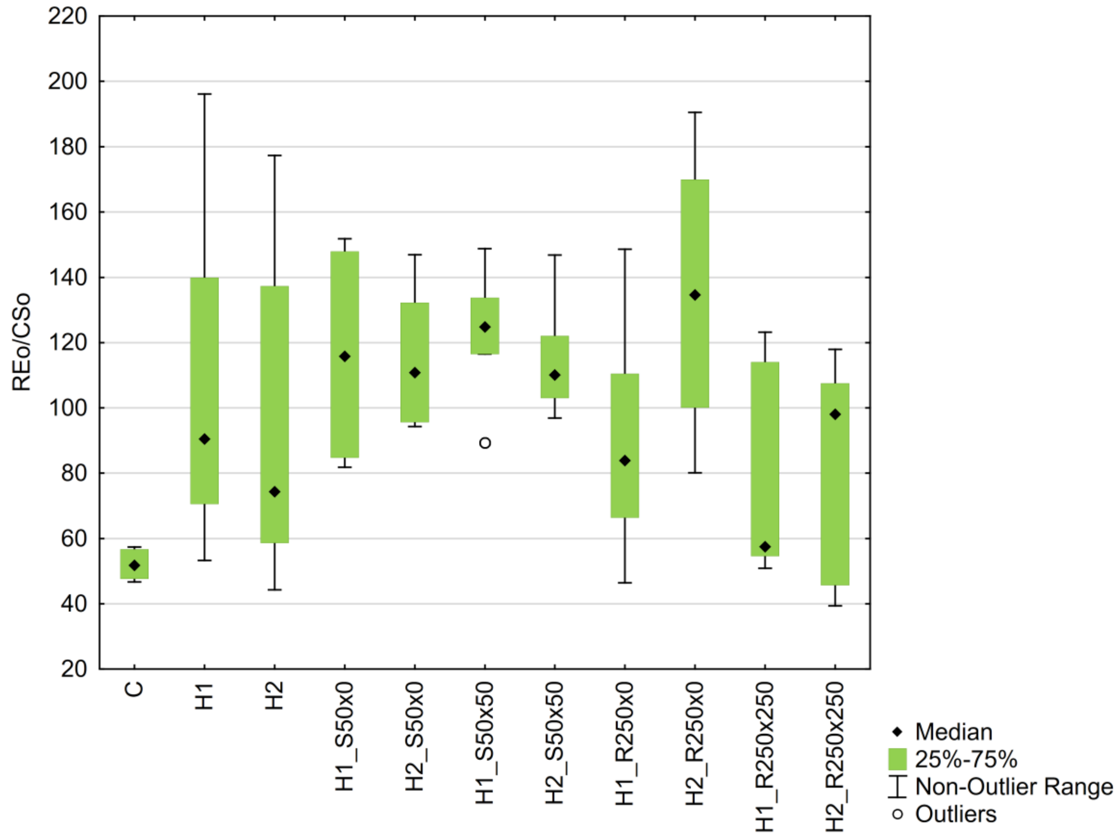

v) REo/CSm

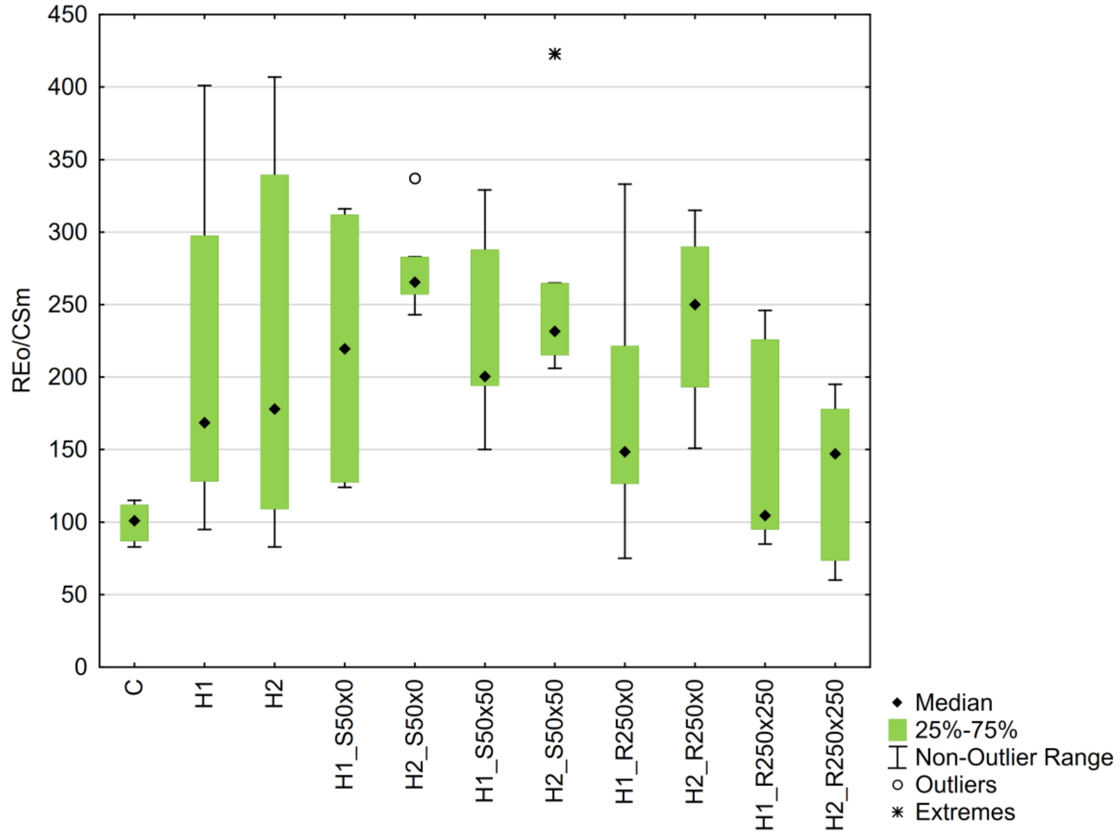

w) PI abs

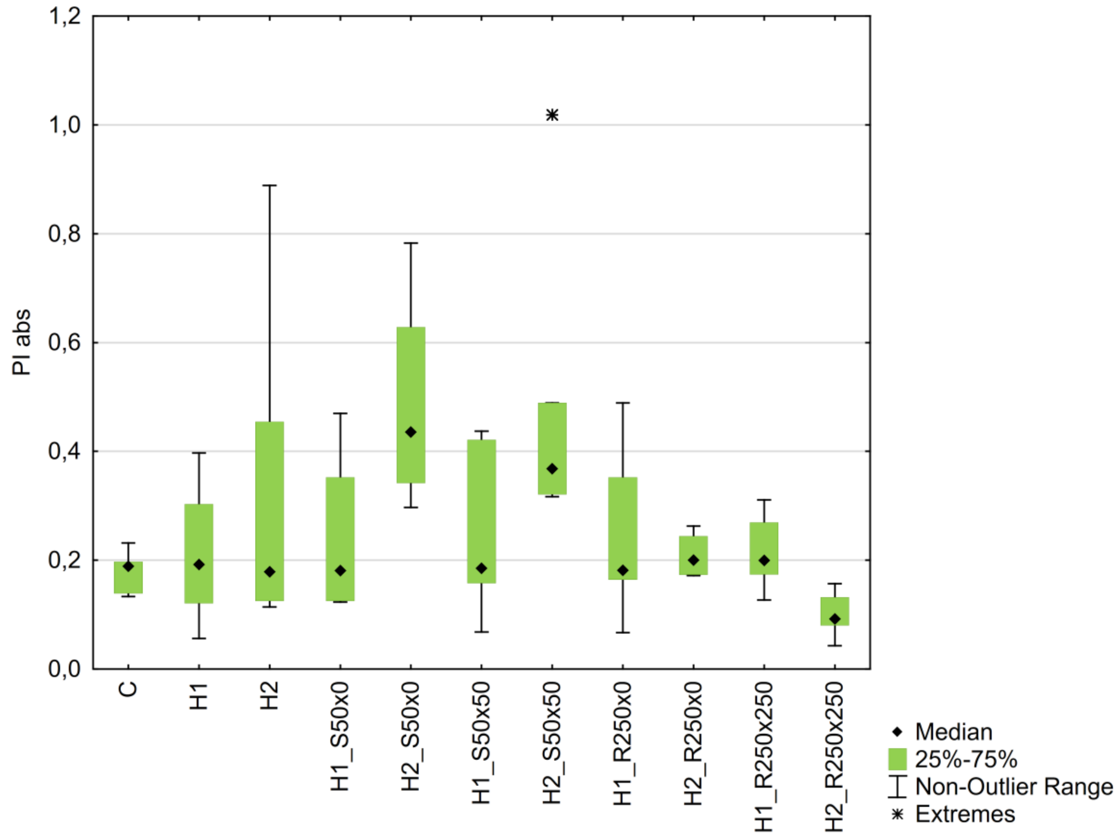

x) REo/RC

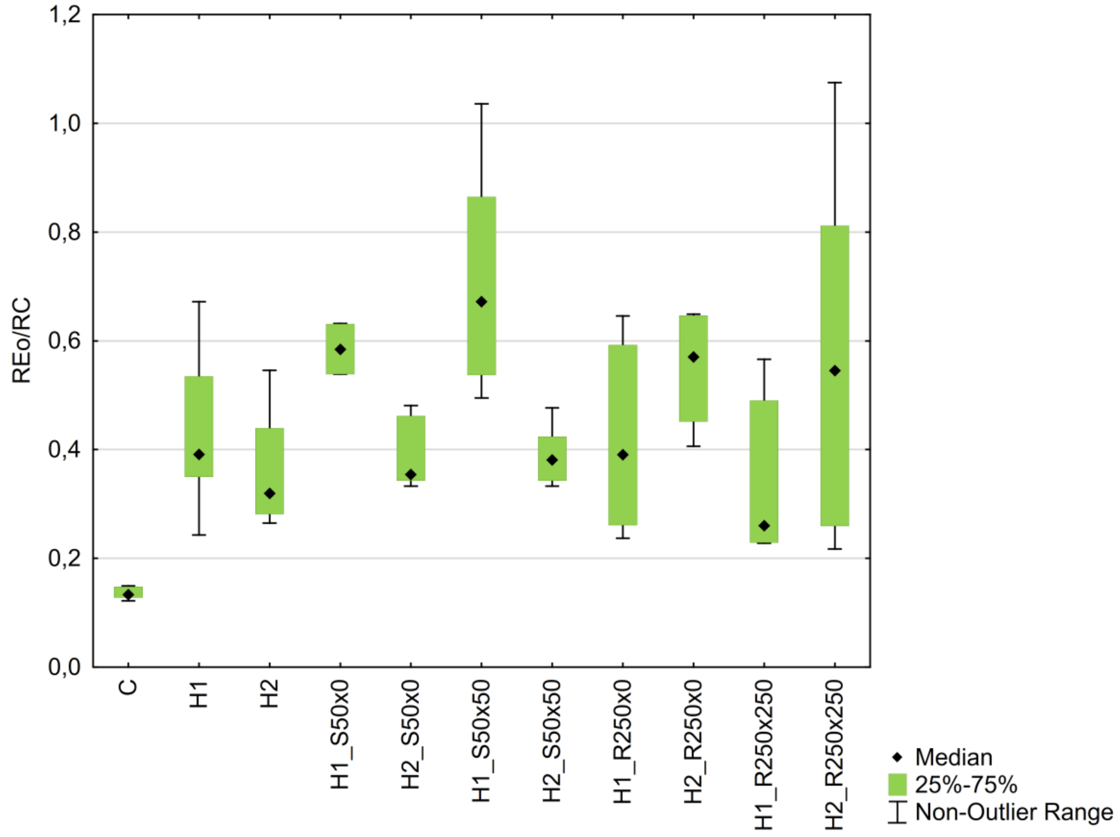

Supplement: Supplementary file 1 [file plants-09-00194-s001.zip › Supplementray material/S2.pdf]
