# Supplementary material for: Physiological Response of Miscanthus x giganteus to Plant Growth Regulators in Nutritionally Poor Soil
Source: Plants (Basel). 2020 Feb 5;9(2):194. doi: 10.3390/plants9020194 (PMC7076640; doi:10.3390/plants9020194)

Figure S4: Average month temperatures, precipitation and light period in Ústí nad Labem in 2017 (source: Czech hydrometeorological institute, http://portal.chmi.cz/historicka-data/pocasi/mesicni-data#)

a) Average month temperature

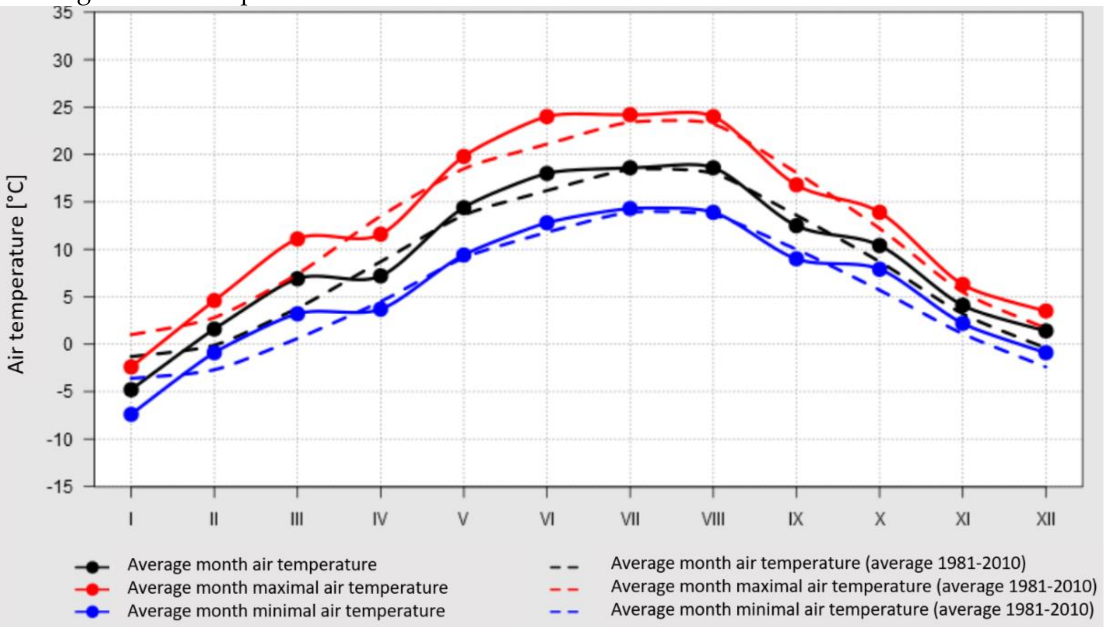

b) Average month precipitation

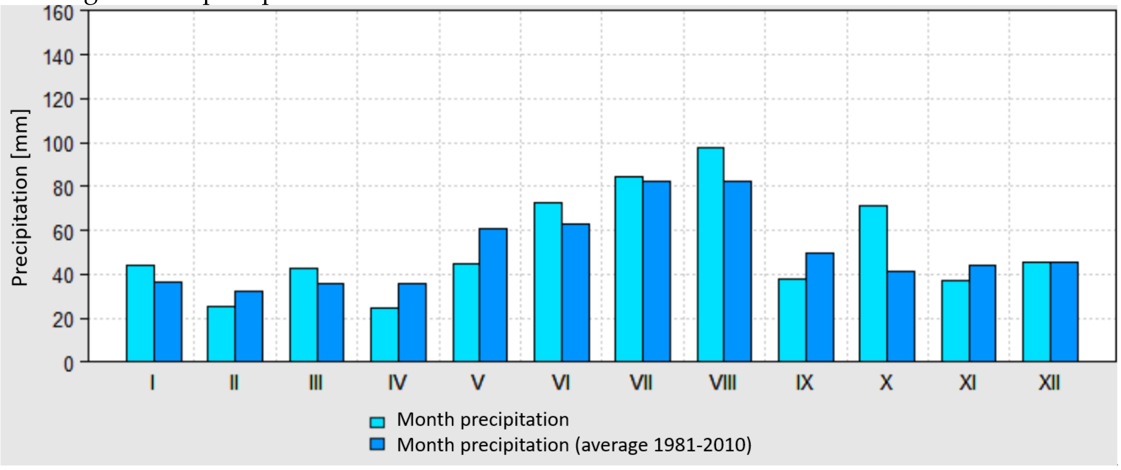

c) Average month light period

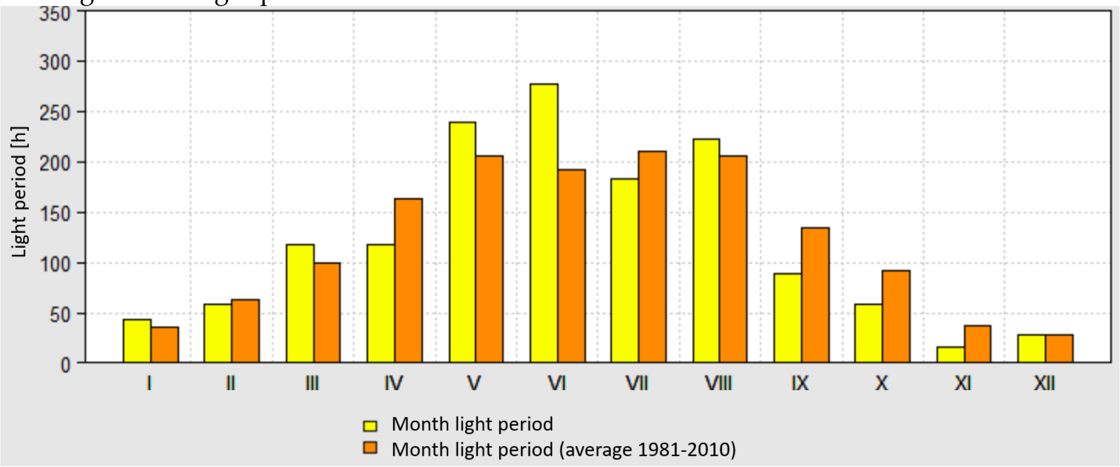

Supplement: Supplementary file 1 [file plants-09-00194-s001.zip › Supplementray material/S4.pdf]
