# Supplementary figures and images for: Physiological Response of Miscanthus x giganteus to Plant Growth Regulators in Nutritionally Poor Soil
Source: Plants (Basel). 2020 Feb 5;9(2):194. doi: 10.3390/plants9020194 (PMC7076640; doi:10.3390/plants9020194)

**Figure S3:** Photograph of the experiment

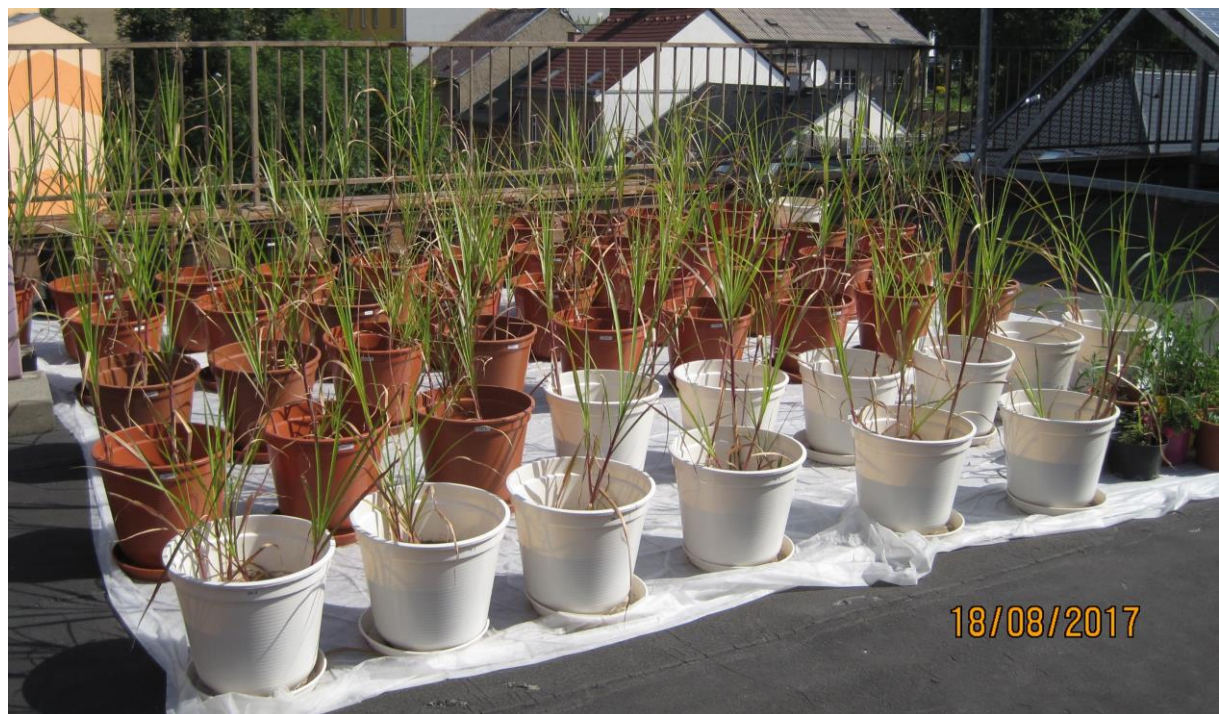

Supplement: Supplementary file 1 [file plants-09-00194-s001.zip › Supplementray material/S3.pdf]
